# Oral Human Papillomavirus Benign Lesions and HPV-Related Cancer in Healthy Children: A Systematic Review

**DOI:** 10.3390/cancers15041096

**Published:** 2023-02-08

**Authors:** Federica Di Spirito, Giuseppe Pantaleo, Maria Pia Di Palo, Alessandra Amato, Annunziata Raimondo, Massimo Amato

**Affiliations:** 1 Department of Medicine, Surgery and Dentistry, University of Salerno, 84084 Salerno, Italy; 2 Department of Neuroscience, Reproductive Science and Dentistry, University of Naples Federico II, 80131 Naples, Italy

**Keywords:** human papillomavirus, HPV, oral lesions, oral manifestations, oral signs, child, children, pediatric, infant, adolescent, teenager, young, verruca vulgaris, squamous cell papilloma, condyloma acuminata, focal epithelial hyperplasia, oral squamous cell carcinoma

## Abstract

**Simple Summary:**

The present systematic review aimed to gain deeper insight into the epidemiology, clinical presentation, and histopathology of HPV-related benign and malignant lesions of the oral mucosa in pediatric patients to improve the multidisciplinary preventive and therapeutic management of oral and general healthcare. The emerging role of HPV in oral carcinogenesis in pediatric subjects, along with benign oral mucosal lesions and asymptomatic infections, brings HPV vaccination to the forefront specifically for this age group.

**Abstract:**

The present systematic review aimed to assess the prevalence of oral HPV-related lesions, categorized as benign (verruca vulgaris “VV”, squamous cell papilloma “SP”, condyloma acuminata “CA”, and focal epithelial hyperplasia “FEH”) and malignant (oral squamous cell carcinoma “OSCC”), in descending order of occurrence in pediatric subjects (≤18 years of age). The secondary objectives were to evaluate the frequency and types of oral lesions described in relation to HPV genotypes and the HPV vaccine type (if any). The study protocol, compliant with the PRISMA statement, was registered at PROSPERO (CRD42022352268). Data from 60 studies, of which quality was assessed using the ROBINS-I tool, were independently extracted and synthesized. Along with seven poorly described benign HPV-related oral lesions that could not be categorized, a total of 146 HPV-related oral lesions, namely 47.26% (*n* = 69) VV, SP, and CA, 51.37% (*n* = 75) FEH, and 1.37% (*n* = 2) OSSC, were diagnosed in 153 pediatric subjects (M:F ratio = 1:1.4) with a mean age of lesion onset of 8.46 years. The viral genotypes detected were HPV-13 (30.61%), -6 (20.41%), -11 (16.33%), HPV-2 (12.24%), -32 (10.20%), -57 (6.12%), and -16 (4.08%). No HPV vaccination was reported in any case. Further studies should be conducted to evaluate the prevalence of HPV-related benign and malignant lesions and the potential role of HPV and associated vaccination in oral carcinogenesis in pediatric subjects.

## 1. Introduction

Human papillomavirus (HPV) is associated with benign and malignant diseases of various locations, mainly affecting the skin and genital and oral mucosa [1]. In detail, the benign HPV-related lesions of the oral mucosa recognized by the World Health Organization are squamous cell papilloma (SP), condyloma acuminatum (CA), verruca vulgaris (VV), and focal epithelial hyperplasia (FEH) [2].

Of the approximately 100 different HPV genotypes that have been identified, 25 are generally associated with oral lesions, including HPV-1, -2, -3, -4, -6, -7, -10, -11, -13, -16, -18, -31, -32, -33, -35, -40, -45, -52, -55, -57, -58, -59, -69, -72, and -73 [3], with low-risk HPV-6 and 11 being the most commonly detected [2]. 

In particular, HPV-6 and HPV-11 are frequently found in squamous cell papillomas [3], HPV-6 and HPV-11 in condyloma acuminata, HPV-2 followed by HPV-57 [3], -4, and -40 [2] in vulvar warts, and HPV-13 and -32 in FEH [3]. 

HPV is also considered an independent risk factor for benign and malignant tumors [1], and an increased incidence of HPV-positive oropharyngeal cancers has been recorded in the last decades [4]. Although the role of HPV in oral squamous cell carcinoma (OSCC) has not been fully elucidated [4], to date, HPV-related oral carcinomas, in which HPV-16 and HPV-18 are the most commonly associated genotypes [5,6], are estimated to account for a smaller proportion (3%) [7] than oral carcinomas attributable to other risk factors, such as tobacco and alcohol [6,8]. 

However, while benign and malignant oral HPV-related lesions in adults have been extensively described in the literature, available data on the pediatric population (≤18 years of age) remain sparse, heterogeneous, and characterized by low evidence, as they are mainly case reports and series. 

Moreover, the emerging role of HPV in oral carcinogenesis in pediatric subjects [9], along with benign lesions and asymptomatic oral mucosal infections, brings to the forefront HPV vaccination specifically for this age group. Indeed, three types of HPV vaccines are currently available: Gardasil (Quadrivalent; Merck & Co., Kenilworth, NJ, USA) against HPV-6, -11, -16, and -18 genotypes, Gardasil9 (Nonavalent; Merck & Co, Kenilworth, NJ, USA) against HPV-6, -11, -16, -18, -31, -33, -45, -52, and -58 genotypes, and Cervarix (Bivalent; GSK, Brentford, UK) against HPV-16 and -18 [10]. 

The Centers for Disease Control and Prevention have developed new recommendations for routine HPV vaccination at ages 9 to 12 years to maximize vaccine effectiveness and increase the number of cancers prevented [11]. 

In light of these considerations, the present systematic review aimed to assess the prevalence of oral HPV-related lesions in pediatric subjects (≤18 years of age), categorize them as benign and malignant, and rank them in descending order of occurrence. The secondary objectives were to evaluate the frequency and type of oral lesions described in relation to HPV genotypes and the HPV vaccine type (if any).

## 2. Materials and Methods

### 2.1. Study Protocol

The study protocol currently used was developed in accordance with the Preferred Reporting Items for Systematic Reviews and Meta-analyses (PRISMA) statement [12] prior to the literature search and data analysis and focused the research questions on the prevalence, clinical presentation, and histopathology of oral HPV-related benign and malignant lesions in pediatric subjects [13].

The formulation of the search question and strategy and criteria for study selection were developed using the PECO model [14].

The study question focused on the following:

P—pediatric subjects (≤18 years of age)
E—human papillomavirus (HPV) infection
C—none or subjects with and without an HPV vaccine and the type
O—oral lesions.

### 2.2. Search Strategy

Case reports, case series, and observational studies in English on oral HPV-related lesions in pediatric subjects (≤18 years of age) were searched electronically in the Scopus, MED-LINE/PubMed, and BioMed Central databases, without filters, by two independent reviewers (F.D.S., M.P.D.P.) until 11 August 2022, using the following appropriate keywords combined with Boolean operators:

1. Human papillomavirus OR HPV

AND

2. Oral lesions OR oral manifestations OR oral signs

AND

3. Children OR pediatric OR child OR infant OR adolescent OR teenager OR young.

### 2.3. Study Selection and Eligibility Criteria

The collected citations were recorded, duplicates were eliminated, and the titles and abstracts obtained were independently screened by two reviewers (F.D.S., M.P.D.P.). The full texts of potentially relevant papers and ambiguous abstracts were reviewed independently by the same authors (F.D.S., M.P.D.P.), who resolved disagreements through discussion and consensus and, if necessary, with the involvement of a third reviewer (G.P.).

An additional manual search was also performed on the reference lists of articles under consideration; relevant titles and abstracts were screened, and full texts were reviewed as described above. If full texts were missing, study authors were contacted.

Inclusion criteria were as follows: case reports, case series, cross-sectional, case-control, prospective, and retrospective studies accepted and published in English between 1970 and 11 August 2022, without limitations of sample size or gender, describing HPV-related oral lesions confirmed via histopathologic analysis (with or without PCR confirmation), excluding FEH cases diagnosed clinically only.

Exclusion criteria were as follows: in vitro and preclinical in vivo studies, systematic and narrative reviews, conference papers, oral communications, and books/chapters; participants ≥ 18 years of age and HIV-positive subjects; normal oral mucosal variations, likely preexisting, and self-reported oral mucosal lesions. 

### 2.4. Data Extraction and Collection

Two reviewers (M.P.D.P., F.D.S.) extracted data independently, and a third reviewer (G.P.) was consulted in cases of discrepancies. A standardized data extraction form was used, developed under models proposed for intervention reviews of RCTs and non-RCTs [13].

The following data meeting eligibility criteria were collected from each study included in this systematic review:

▪ First author, year, journal, funding, study quality;
▪ Design and number of studies reported; sample size, gender ratio, mean age, comorbidities of the population investigated; HPV vaccine (if any; targeted HPV genotypes);
▪ Total number or prevalence of pediatric subjects with oral HPV-related lesions diagnosed through a clinical examination confirmed based on histopathologic analysis (except for FEH) with/without PCR confirmation;
▪ Macroscopic and microscopic features of oral lesions categorized as benign and malignant oral lesions [15];
▪ Definitive diagnosis, diagnostic procedures, treatment, and progression of the oral lesions;
▪ HPV genotypes associated with oral lesions.

### 2.5. Data Synthesis

A narrative synthesis was performed that focused on the reported characteristics of the pediatric population investigated, HPV exposure and related vaccination (if any), and oral outcomes.

Data from the included studies were qualitatively synthesized through descriptive statistical analysis using the Statistical Package for the Social Sciences (SPSS, version 25.0, Armonk, NY, USA)/Microsoft Excel Software 2019, Microsoft Corporation, Redmond, WA, USA):

➢ Estimating the prevalence of HPV-related lesions in pediatric subjects diagnosed via clinical examination and confirmed based on histopathologic analysis;
➢ Categorizing oral HPV-related lesions into benign, potentially malignant, and malignant lesions and ranking them in descending order of occurrence;
➢ Evaluating the frequency of oral lesions categorized as benign, potentially malignant, and malignant in pediatric subjects
➢ Calculating the frequency of HPV genotype detected in oral lesions;
➢ Comparing the frequency and type of oral lesions in subjects with and without HPV vaccination
➢ Evaluating the oral lesions in relation to the HPV vaccine type.

### 2.6. Quality Assessment 

The risk of bias in the studies included in this systematic review was assessed by three independent reviewers (F.D.S, M.P.D.P., G.P.) using the Risk of Bias Instrument for Non-randomized Studies of Exposures [16], which is a modified version of the ROBINS -I (Risk Of Bias In Non-randomized Studies of Interventions) instrument [17], which takes into account the bias due to confounding, participant selection, intervention classification, deviations from planned interventions, missing data, selection of reported outcomes, and bias in outcome measurement. 

The risk was classified as “low” or “moderate” if the risk of bias was low or low or moderate in all domains, respectively. A “serious” risk of bias was defined if a serious risk of bias was identified in at least one domain, but a critical risk of bias was not identified in any domain. The study was considered “critical” if the risk of bias was found to be critical in at least one area.

## 3. Results

### 3.1. Study Selection

Electronic searches retrieved a total of 83 records, 47 from MEDLINE/PubMed, 2 from Scopus, and 34 from BioMed Central databases; 3 duplicates were subsequently removed. The remaining 80 titles/abstracts were screened, and 37 were excluded because: 21 did not focus on HPV (6 focused on HIV); 5 reported oral HPV-related infection or lesions in adult women; 5 were in vitro studies; 2 did not report oral lesions (extra-oral lesions only); 2 were questionnaires assessing HPV knowledge; 1 was an animal study; 1 assessed oral HPV infection without associated oral lesions.

Of the 43 abstracts that were relevant to the present systematic review and met the eligibility criteria, the full texts were screened, and an additional 35 articles were excluded, specifically because: 5 studies did not report oral lesions; 15 did not involve pediatric patients, or it was not possible to extract data from them; 10 involved >18-year-old subjects; 2 described oral lesions that were not HPV-related; 3 were reviews. 

A total of eight studies [18,19,20,21,22,23,24,25] were found in the electronic search and included in this systematic review.

Fifty-two additional records [1,2,26,27,28,29,30,31,32,33,34,35,36,37,38,39,40,41,42,43,44,45,46,47,48,49,50,51,52,53,54,55,56,57,58,59,60,61,62,63,64,65,66,67,68,69,70,71,72,73,74,75] that were manually retrieved through the reference lists of the eight articles already included in the present systematic review [16,17,18,19,20,21,22,23] and met the currently applied eligibility criteria were presently included. 

**Figure 1:**
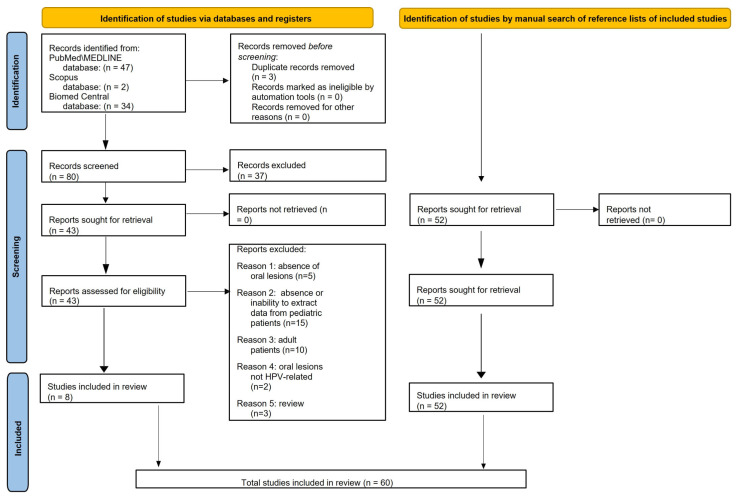
PRISMA 2020 flow diagram for electronically and manually retrieved records.

Finally, the present systematic review included 60 articles on oral HPV-related lesions in pediatric subjects (≤18 years of age) (Figure 1).

Data from 60 studies describing oral HPV-related lesions diagnosed through clinical examination and confirmed based on histopathologic analysis in pediatric subjects were extracted and synthesized.

Of the 60 included studies, 36 were case reports, 19 were case series, 3 were retrospective, and 2 were prospective studies involving a total of 153 cases diagnosed with HPV-related oral lesions in pediatric (≤18 years of age) subjects.

The sources, methods, and qualitative synthesis of oral outcomes from the included studies were pooled. Only data that met eligibility criteria were extracted and analyzed; therefore, data from individuals who were >18-years-old were not detailed.

### 3.2. Studies Reporting Pediatric Cases Diagnosed with Oral Papilloma, Verruca Vulgaris, or Condyloma Acuminata

Table 1 summarizes data from included studies describing pediatric (≤18 years of age) cases diagnosed with oral papilloma, verruca vulgaris, or condyloma acuminata.

#### 3.2.1. Oral Papilloma, Verruca Vulgaris, or Condyloma Acuminata: Case Characteristics 

A total of 76 pediatric subjects were diagnosed with oral HPV-related lesions, which were divided into 37 papillomas [17,20,21,23,25,28,29,30,31,33,35,40,41,42,45,46], 19 verrucae vulgaris cases [2,22,24,36], and 13 condyloma acuminata cases [2,26,27,32,34,37,38,43,44]. 

The study population included 35 males and 33 females between the ages of 1.5 and 18 years, with a mean age of 8.9. However, the gender and age of six subjects were not reported [40]. 

Country of origin or ethnicity was reported for 28 patients, as follows: 7 Black [21,23]; 2 Latino [23]; 16 Caucasian [23,27,38,43,44,46], including 1 from Denmark [27]; 1 from Somalia [37]; 1 from North Kerala [41]. 

Patients from 11 studies had no comorbidities [17,20,25,26,28,29,31,41,43,44,45], while in one case the patient had impetigo and gonorrhea [34] and in another case, acute respiratory illness [43].

Associated treatments for comorbidities were antibiotics [34], aciclovir [43], and podophyllin for skin warts [44]. 

HPV exposure was associated with the following: suspected abuse [26,34,46]; sexual abuse in three cases [38,43]; vaccination in two cases [39]; vertical transmission in eight cases [40]. Relatives with similar HPV-related lesions on any body site were as follows: the father [26], the mother [43], and a foster child [37]; no information was provided for four reported cases [17,34,41,43].

The mean time to the onset of oral lesions was 11.4 months and ranged from 14 days to 7 years. Only one study [28] reported that the patient had not received an HPV vaccine.

#### 3.2.2. Oral Papilloma, Verruca Vulgaris, or Condyloma Acuminata: Macroscopic and Microscopic Features, Extra-Oral Involvement, HPV Genotype 

Macroscopic features were reported in 22 [2,17,20,25,26,28,29,30,31,32,33,34,35,37,38,39,40,41,42,43,44,45], while microscopic ones were reported in 20 studies [2,17,20,21,24,25,28,29,30,31,32,33,34,35,37,38,39,41,42,44], respectively. 

HPV-related oral manifestations occurred as a single lesion in 22 cases [2,20,25,26,29,30,31,32,33,35,40,43,45,46] and as multiple lesions in 10 cases [17,28,34,37,38,41,42,43,44], with a unilateral distribution in 18 cases [25,26,29,30,31,32,33,34,35,38,41,42,45,46], bilateral distribution in 11 cases [2,17,28,37,40,43], and asymmetric pattern in 2 cases [28,37]; the lesions tended to coalesce in 1 reported case [44]. 

The affected sites were as follows: lower lip (*n* = 8) [24,28,30,34,37,41] and upper lip (*n* = 9) [17,24,26,37,39,41,44]; palate (*n* = 13) [20,31,35,38,39,40,42,43]; tongue (*n* = 9) [17,30,36,37,38,42,43,44]; commissures (*n* = 6) [24,28,30,39,43]; cheeks (*n* = 5) [17,28,37,43]; gingiva (*n* = 6) [2,17,21,27,29,36]; uvula (*n* = 3) [25,33,45]; tonsils (*n* = 2) [46]; vermilion (*n* = 2) [2]; anterior faucial columns (*n* = 2) [27,40]; retromolar area (*n* = 1) [17]; vestibule (*n* = 1) [42]; N\D oral mucosa (*n* = 1) [36].

Thirteen patients had no extra-oral involvement on examination [17,27,31,35,36,37,42,43], while eleven others also had extra-oral involvement, namely: warts on the hand or fingers in six cases [27,28,36,39]; genital warts in two cases [26,44]; skin warts in one case [34]; warts on the forehead in one case [21]; laryngeal papillomas in one case [46] 

HPV genotypes detected in oral lesions were as follows: HPV-2 in six cases [24,36]; HPV-6 in nine cases [2,21,26,27,28,37,46]; HPV-11 in seven cases [2,26,28,37,46]; HPV-57 in three cases [36]; HPV-16 in two cases [17,40]; HPV-32 in one case [41]. In 16 cases, the viral genotypes studied were not further described [2,21,24,27,36,38,39].

#### 3.2.3. Oral Papilloma, Verruca Vulgaris, or Condyloma Acuminata: Definitive Diagnosis, Diagnostic Procedure(s), Therapy, Progression

The diagnostic procedures performed included the following: biopsy (*n* = 48) [17,20,21,23,26,27,28,29,30,31,32,33,35,36,37,38,39,40,41,42,44]; in situ hybridization (*n* = 28) [2,21,22,24,26,27,28,36,37,40,43,46]; immunoperoxidase assays for viral antigens (*n* = 2) [21]; PCR analysis (*n* = 3) [17,40,41]; immunohistochemistry for the p53 protein (*n* = 1) [30]; cytologic scrapes (*n* = 8) [40]; vaginal culture (*n* = 1) [34] testing positive for *Neisseria* (*N.*) *lactamina*, *N. meningitides*, *N. gonorrhoeae*; swabs (*n* = 3) [43]; radiographs (*n* = 1) [45]; direct laryngoscopy (*n* = 1) [45]; serological tests (*n* = 5) [17,37,43] yielding negative results for HIV in four cases [17,43], *Treponema pallidum* and HBV in three cases [43], antisyphilis antibodies [17]. The following therapies were performed: Electrocautery in 2 cases [25,41]; CO_2_ laser surgery in 2 [28]; laser surgery in 7 [20,29,43]; excisional biopsy in 10 [31,32,33,34,40,42,44,45]; oral surgery in 1 [17]; cimetidine in 1 [43]; interferon alpha-2a in 1 [37]; podophyllin in 2 [37,44].

### 3.3. Studies Reporting Pediatric Cases Diagnosed with Focal Epithelial Hyperplasia

Table 2 shows data from retrieved studies describing pediatric (≤18 years of age) cases diagnosed with oral focal epithelial hyperplasia.

#### 3.3.1. Focal Epithelial Hyperplasia: Case Characteristics 

Seventy-five pediatric subjects were diagnosed with focal epithelial hyperplasia [2,18,21,29,49,50,51,52,53,54,55,56,57,58,59,60,61,62,63,64,65,66,67,68,69,70,71,72,73,74,75]. Two studies [56,62] included in this systematic review described cases of FEH in adult patients who reported having the condition since childhood; data from these two studies are shown in Table 2 but were not included in the data analysis. The study population included 18 males and 45 females aged 0–18 years, with a mean age of 9.97 years. However, gender was not specified for 10 subjects [21]. 

Country of origin or ethnicity was reported for 52 patients, specifically as follows: 2 Hispanic [50,69]; 1 Caucasian [67]; 1 Black [74]; 1 African [51]; 1 Greenlandic Eskimo [29]; 9 Venezuelan [24]; 2 Dutch [72]; 1 Ecuadorian [52]; 6 Brazilian [53,63]; 2 Mexican [54]; 8 Iranian [57,64]; 1 Moroccan [61]; 1 Algerian [61]; 1 Turkish [68]; 1 Libyan [58]; 3 Australian [59]; 1 Chinese [60]; 6 Ghanaian [66]; 2 Ikalouit [73]; 2 Guyanese [71]. 

HPV exposure was associated with the following: 1 suspected abuse [55]; 1 horizontal transmission [71]. Relatives with similar HPV-related lesions anywhere on the body were as follows: siblings in 13 cases [24,53,55,58,59,61,71,72,73]; mother in 2 cases [59,71]; father [73] and cousin [58] in 1 case, each; none in 7 reported cases [52,60,63,66,74,75].

The median time to onset of oral lesions was 19.9 months and ranged from 1 month to 8 years. No study reported whether or not patients were vaccinated against HPV.

#### 3.3.2. Focal Epithelial Hyperplasia: Macroscopic and Microscopic Features, Extra-Oral Involvement, HPV Genotype 

Macroscopic and microscopic features were reported in 27 [18,24,49,50,52,53,54,55,57,58,59,60,61,63,75] and 24 studies, respectively [18,24,50,51,52,54,55,57,58,59,60,61,63,64,65,66,67,68,69,70,72,74,75]. 

Oral FEH occurred in 54 pediatric cases [2,18,24,49,50,51,52,53,54,55,57,58,59,61,63,64,65,66,67,68,69,70,71,72,73,74,75], with a unilateral distribution in 4 cases [64,69,71], bilateral distribution in 10 cases [18,54,60,61,64,70,71,73], asymmetric pattern in 13 cases [51,52,55,57,58,59,65,75]; lesions tended to coalesce in 5 cases [49,57,61,71,75].

The affected sites were as follows: lips (*n* = 51) [2,18,24,29,49,50,51,52,53,54,55,57,58,59,60,61,64,65,66,67,68,69,70,72,74,75], specifically lower lip (*n* = 21) [18,29,49,51,53,54,55,58,59,64,65,68,72,75] and upper lip (*n* = 15) [24,51,53,54,58,64,67,68,72,75]; cheeks (*n* = 30) [18,49,50,52,53,54,58,60,61,64,66,67,68,69,70,71,72,73,74,75]; tongue (*n* = 19) [24,49,51,52,53,54,55,59,61,65,66,71,72,73]; commissures (*n* = 9) [58,66,68,72]; gingiva (*n* = 8) [49,50,54,61,63,66,72]; palate (*n* = 5) [61,72,75]; the floor of mouth (*n* = 3) [54,66,72]; vermillion (*n* = 3) [58]; vestibule (*n* = 1) [70]; anterior faucial columns (*n* = 1) [72]; retromolar area (*n* = 1) [74]. 

Nine patients did not show extra-oral involvement on examination [51,52,54,60,65,67,70,73], while nine also showed extra-oral involvement; notably, skin warts were described in 12 cases [29,58,61,68], and 3 of them were located on the hand [29,58,61]. 

The HPV genotypes detected were as follows: HPV-13 in 15 cases [24,29,50,52,53,54,57,58,60,63,68,71]; HPV-32 in 4 cases [51,57,65,67]; HPV-6 and 11 in 1 case [2].

#### 3.3.3. Focal Epithelial Hyperplasia: Definitive Diagnosis, Diagnostic Procedure(s), Therapy, Progression

In five cases, no therapies were performed [50,57,60,66,67]. The treatments that were performed in the remaining cases were as follows: TCA in 12 cases [21,75]; electrocautery [61,75], laser surgery [52,54,65], and interferon alpha-2b [49] in 3 cases each; CO_2_ laser surgery [18,49]; shaving [61], excisional biopsy [63,69], cryotherapy [55,75], and levamisole [49,75] in 2 cases each; Quantum Molecular Resonance Scalpel [70], oral surgery [54], acitretin [49], podophyllin [55], vitamin A, and imiquimod [75] in 1 case each. 

Regarding the progression of oral FEH lesions: in 14 cases, lesions healed after an average of 15.95 months, ranging from 14 days to 3 years; in 17 cases, no recurrence occurred after an average of 11 months, with a follow-up ranging from 1.5 months to 1 year. Improvement was noted in seven cases after an average of 18.4 months, ranging from 1 month to 5 years. Recurrence occurred in two cases, after six months in 1 case, while deterioration was declared in 1 case and no improvement occurred in 9 cases with an average follow-up time of 27 months.

### 3.4. Studies Reporting Pediatric Cases Diagnosed with Oral Squamous Cell Carcinoma

Table 3 summarizes data from identified studies characterizing pediatric (≤18 years of age) cases diagnosed with oral squamous cell carcinoma.

#### 3.4.1. Oral Squamous Cell Carcinoma: Case Characteristics 

A total of two pediatric subjects were diagnosed with oral malignant HPV-related lesions categorized as oral squamous cell carcinoma (OSCC) [1,20].

The study population included two males aged 5 to 8 years, with a mean age of 6.5 years, one of whom was of Caucasian origin [20], and both were without comorbidities. 

In one case, HPV exposure through self-vaccination was suspected [18], which was supported by evidence that his two brothers also had HPV-related lesions [18].

#### 3.4.2. Oral Squamous Cell Carcinoma: Macroscopic and Microscopic Features, Extra-Oral Involvement, HPV Genotype

Macroscopic and microscopic features were reported in both studies [1,18], and in two cases, the lesions appeared to be solitary [1,18] and unilateral [1,18] and involved the maxillary ridge [1,18]. 

Extra-oral involvement was described in one case [18] as warts on the hands, chin, philtrum, and commissures. 

The HPV genotype involved was not reported, but p16 positivity was noted in both cases; HPV association with pediatric OSCC was therefore reported based on the p16 status of the lesions. 

#### 3.4.3. Oral Squamous Cell Carcinoma: Definitive Diagnosis, Diagnostic Procedure(s), Therapy, Progression

The diagnostic procedures performed were as follows: two biopsies [1,18]; one periapical radiograph [18]; two CBCT [1,18]; one PCR analysis [18]; one CT [1], and one RMI [1]. 

Therapies performed were as follows: one pharmacologic therapy with antibiotics [18]; one tooth extraction [18]; biopsies [1,18]; and two partial maxillectomies [1,18]. 

In one case, the patient healed after 24 months [1] since the partial maxillectomy, while in one case, the patient had two recurrences [18] and one exacerbation [18] and healed only 4 weeks after the partial maxillectomy [18].

### 3.5. Prevalence of Reported Oral HPV-Related Lesions in Pediatric Subjects

**Figure 2:**
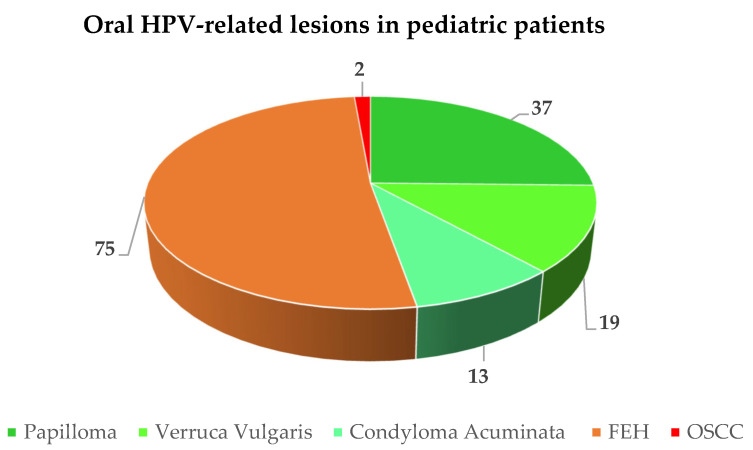
Frequency of reported oral HPV-related lesions in pediatric patients.

A total of 146 HPV-related oral lesions were diagnosed in 153 pediatric subjects (M:F ratio = 1:1.4) with a mean age of 8.46 years, including 47.26% (*n* = 69) verrucae vulgaris, squamous cell papillomas, and condyloma acuminata, 51.37% (*n* = 75) FEH, and 1.37% (*n* = 2) OSSC (Figure 2). In addition, seven poorly described benign HPV-related oral lesions were reported but could not be categorized.

On average, patients visited a physician 15.65 months after the appearance of oral lesions, and in no case was HPV vaccination indicated. 

The most frequently affected sites were as follows, in descending order: lips (36.6%) in 74 cases (upper lip was reported in 29 and lower lip in 24 cases), followed by cheeks (17.3%) in 35 cases, tongue (13.9%) in 28 cases, palate (8.9%) in 18 cases, labial commissures (7.4%) in 15 cases, gingiva in 14 cases, vermilion (2.5%) in 5 cases, anterior faucial columns (1.5%), uvula (1.5%), and floor of mouth (1.5%) in 3 cases, tonsils (0.99%), the vestibule (0.99%) and alveolar ridge (0.99%) in 2 cases; N\D oral mucosa in 1 (0.5%).

### 3.6. HPV Genotypes Detected in Oral HPV-Related Lesions in Pediatric Subjects

**Figure 3:**
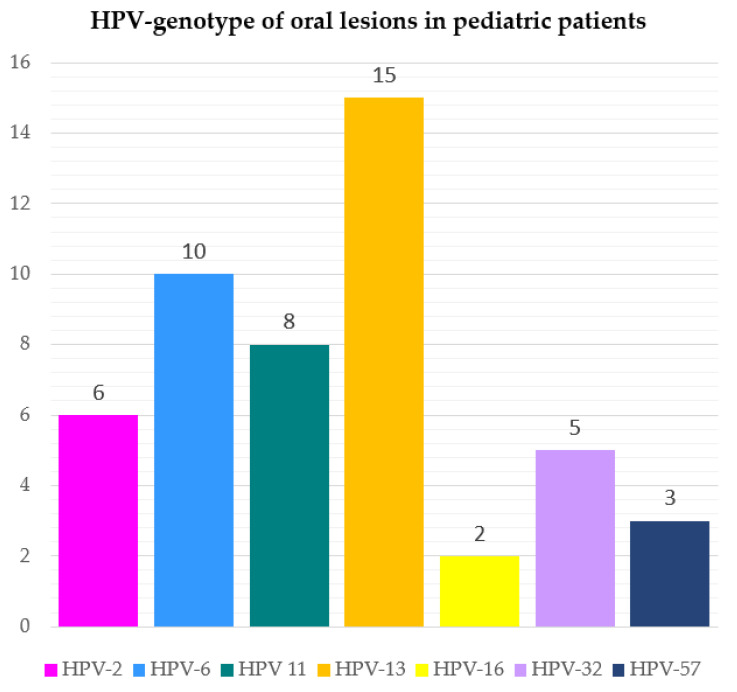
Frequency of viral genotypes detected in HPV-related oral lesions in pediatric patients.

**Figure 4:**
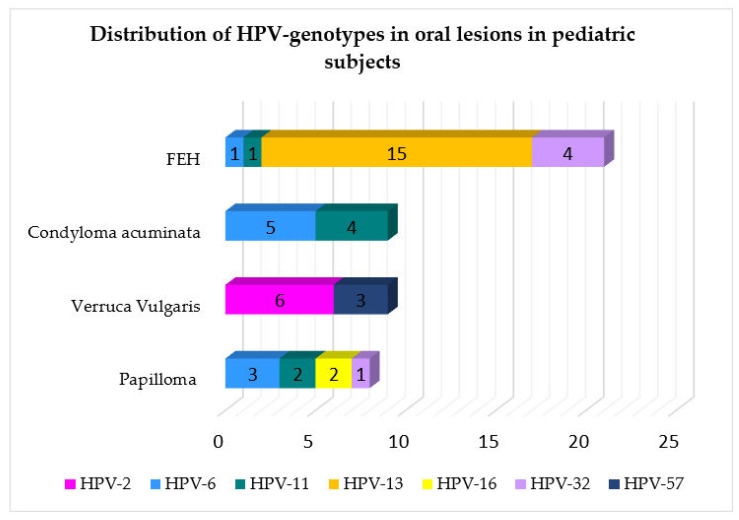
Distribution of HPV genotype in relation to the type of oral lesions reported in pediatric patients.

The HPV genotypes detected were as follows: HPV-13 in 15 cases (30.61%); HPV-6 in 10 cases (20.41%); HPV-11 in 8 cases (16.33%); HPV-2 in 6 cases (12.24%); HPV-32 in 5 cases (10.20%); HPV-57 in 3 cases (6.12%); HPV-16 in 2 cases (4.08%), as shown in Figure 3 and mapped in Figure 4 in relation to the type of oral lesion.

### 3.7. Quality Assessment 

The risk of bias of the non-randomized studies included in the present systematic review is reported in Table 4.

## 4. Discussion

The present systematic review aimed to assess the prevalence of oral HPV-related lesions in pediatric subjects (≤18 years of age), categorize them as benign and malignant, and rank them in descending order of occurrence. The secondary objectives were to evaluate the frequency and types of oral lesions described in relation to HPV genotypes and the HPV vaccine type (if any).

Sixty studies were considered, and along with seven poorly described benign HPV-related oral lesions that could not be categorized, a total of 146 HPV-related oral lesions, namely 47.26% (*n* = 69) VV, SP, and CA, 51.37% (*n* = 75) FEH, and 1.37% (*n* = 2) OSSC, were diagnosed in 153 pediatric subjects (M:F ratio = 1:1.4) with a mean age of lesion onset of 8.46 years. The viral genotypes detected were HPV-13 (30.61%), -6 (20.41%), -11 (16.33%), HPV-2 (12.24%), -32 (10.20%), -57 (6.12%), and -16 (4.08%). No HPV vaccination was reported in any case. 

### 4.1. Oral Benign HPV-Related Lesions in Pediatric Subjects: Verruca Vulgaris, Squamous Cell Papilloma, and Condyloma Acuminatum 

The investigated population with oral benign HPV-related proliferation was almost evenly distributed between the female and male genders, with an M:F ratio = 1.06:1. A similar distribution has been reported for other viral infectious diseases associated with oral manifestations in the pediatric population, such as HIV (M:F = 1:1) [76] and SARS-CoV-2 (M:F = 1.3:1) [77].

The pediatric cases diagnosed with benign oral HPV lesions had a mean age of 8.9 years, slightly lower than the mean age found for FEH (9.97 years), as described later, and lower than the range reported for the highest incidence of verruca vulgaris, between 12 and 16 years of age [74].

In general, HPV-related oral benign lesions are more common in adults [78], similar to the rate of detectable oral HPV infection, which also increases in adulthood [79]. More specifically, detectable oral HPV infection has two peaks in prevalence at ages 30–34 and 60–64 years [79]. It has been hypothesized that the peak at older ages may be related to the normal process of immunosenescence [80], leading to the possible reactivation of latent HPV infections [81]. Nevertheless, benign HPV-related oral lesions have also shown a similar bimodal peak, with condylomata acuminata showing a higher incidence between the third and fourth decades of life and squamous cell papillomas between the third and seventh decades of life [78]. 

The main transmission route for HPV is skin-to-skin or skin-to-mucosa contact [82]. Sexual transmission has also been adequately documented [80]. Accordingly, in the present systematic review, three cases were sexually abused [40,45] and three cases were investigated for suspected sexual abuse [28,36,48]. Horizontal transmission can occur via fomites, fingers, mouth, and skin contact [82]. Most skin warts in children result from horizontal transmission [15]. For oral mucosal lesions, few studies in the present review also investigated the presence of potentially HPV-related lesions on the body in the patient’s family members, and only three cases [28,39,45] were found in at least one family member, suggesting presumed horizontal transmission. In two patients in the present study, self-inoculation was suspected in virgin women and children with genital warts without suspected sexual abuse [82]. Self-inoculation is typical of vulvar warts in children who suck their thumbs or carry their fingers in their mouths [78]. Simultaneous evidence of lesions in the genital area and oral cavity may indicate sexual transmission [78]. Another route of transmission is vertical mother-to-child transmission [82], which is the main route of HPV infection in newborns [15]. Finally, several studies have suggested the possibility of viral transmission through the amniotic fluid or placenta or through contact with the genital mucosa of the mother during delivery [82]. The results of the present study do not suggest a higher prevalence of one route of transmission than the other. However, most of the included studies did not investigate this aspect of infection.

Squamous papillomas are estimated to be the most common benign oral epithelial le-sions in both pediatric and adult populations [78], which is consistent with the present results (*n* = 37). 

Second, in overall frequency, verrucae vulgaris is the predominant cutaneous manifestation of HPV infection, with a total incidence of 10% in children and young adults [74]. Although considered uncommon in the oral cavity [74], VV cases have currently been observed in 19 cases, accounting for 12.42% of all diagnoses.

Condyloma acuminata is more common anogenitally and is the most common manifestation of sexually transmitted infections in the United States and the United Kingdom [74]. It is also estimated that CA cases are not common in the oral cavity [74], which is consistent with the present results showing that CA is the least frequently reported oral manifestation in pediatric subjects (*n* = 13).

Rarely, multiple verrucae vulgaris occurs together in the oral cavity or clusters [74]; the same is true for SPs. Indeed, in the study by Frigerio et al. [83], which examined 205 oral SPs, only four patients had more than two lesions simultaneously.

Despite the fact that CAs are the benign HPV-related oral lesions that most commonly occur as multiple lesions and tend to coalesce and although Panici et al. [84], examining 101 oral Cas, found 61% of patients with more than five lesions, the retrieved data revealed a higher prevalence of single (22 cases) compared with multiple lesions (10 cases), most of which were represented by CA [2,36,39,40,45,46].

The palate and tongue are considered the sites most commonly affected by SPs [78]. VV, on the other hand, occurs most frequently on the labial mucosa and palate [74], whereas CA occurs on the tongue and upper lip [74]. In line with these findings, the results of the present systematic review showed that benign HPV-related oral lesions most frequently affected the lips (*n* = 23; 31.08%), with apparently no preference for the upper or lower lip, palate (*n* = 13; 17.57%), and tongue (*n* = 9; 12.16%).

In the normal oral mucosa, latent infection is most commonly maintained by HPV-6 and -11 [3], and oral squamous cell papillomas are generally associated with specific viral genotypes. Consistent with reported evidence [2,3], findings of the present systematic review pointed out that HPV-6 was the most frequently detected genotype: in SPs, HPV-6 (*n* = 3), followed by HPV-11 (*n* = 2), 16 (*n* = 2), and 32 (*n* = 1); in CA, HPV-6 (*n* = 5) and HPV-11 (*n* = 4); in VV, HPV-2 (*n* = 6) and HPV-57 (*n* = 3).

Only one study [30] reported that the child had not received HPV vaccination, whereas such information was not detailed elsewhere. Especially, in this pediatric population, dentists should make patients and their parents and caregivers aware of the importance of HPV vaccination, even in children who already have infections, regardless of their HPV status. The American Cancer Society (ACS) has developed new recommendations for HPV vaccination that should be routinely administered between the ages of 9 and 12 to achieve the greatest vaccine efficacy and increase the number of cancers prevented [11]. Healthcare providers should encourage vaccination as early as 9 to 10 years of age and if appropriate, advise those who have not been vaccinated or have not completed vaccination that vaccine administration is less effective in reducing cancer risk at an older age [9]. It is therefore crucial that the pediatric population is encouraged to receive HPV vaccination, as vaccines are critical prevention measures, as was also demonstrated during the COVID-19 pandemic, in which vaccines significantly reduced the risk of virus transmission and infection and prevented severe forms of the disease [85].

### 4.2. Oral Benign HPV-Related Lesions in Pediatric Subjects: Focal Epithelial Hyperplasia 

Focal Epithelial Hyperplasia (FEH), also known as Heck’s disease, is an asymptomatic and benign condition caused by HPV-13 and/or -32 genotypes [56,86] that presents in the oral cavity as multiple, well-circumscribed, raised, soft papules, and nodules with the same color as the surrounding oral mucosa [56,87].

FEH can affect children and adults [15] but generally shows a higher prevalence in younger individuals, with the first and second decades of life being most commonly affected [87]. Accordingly, the mean age currently calculated was 9.97 years. These data are consistent with those of Sethi et al. [88], who studied a broader age range and included subjects aged 3 to 92 years with a mean age of 23.1.

The same study [88] recorded a male-to-female ratio of 3:4. The higher prevalence of FEH in females has also been described in other studies [86,88,89] with a ratio of 3:4 to 1:5, which is consistent with the findings of the present systematic review that recorded a male-to-female ratio of 2:5. The reason for the higher incidence of FEH in females is still unclear and may be due to the poor hygienic conditions in which women live in certain ethnic groups [90], especially in some regions with a higher FEH prevalence. 

In fact, there are no accurate data on the prevalence of FEH in the general population, but it is considered a rare condition [15]. According to initial studies, it occurs most frequently in Native Americans, Mexican Indians, South Americans, and Eskimos [15,89]. Accordingly, the data extracted in this systematic review showed that the country of origin from which most cases of FEH were reported in pediatric patients was Venezuela (*n* = 9) [24], followed by Iran (*n* = 8) [57,64], Brazil, and Ghana (*n* = 6) [53,63,66]. Overall, 20 (26.7%) of the 75 FEH cases were registered in South America, an epidemiological finding consistent with the initial estimate of the geographic distribution of FEH [13]. The involvement of the African continent also seems consistent, with 10 (13.3%) of the cases included in this systematic review (Morocco (*n* = 1) [61], Libya (*n* = 1) [58], Algeria (*n* = 1) [59], Africa (*n* = 1) [51]; Ghana (*n* = 6) [66]). In addition, data from the present study also show a high incidence of FEH in Iran, where 8 (10.7%) of the 75 cases occurred [57,64]. This suggests that Heck’s disease is generally rare and likely more common in certain ethnic and racial groups [89] living in more densely populated and developing countries. These contexts are even referred to as ‘prisons’, underscoring the restricted living conditions that place them at high risk for infection [NO_PRINTED_FORM]. Coherently, fewer cases have been diagnosed with oral FEH in Europe and North America, although they have slightly increased between 2001 and 2019 (20 published cases) compared to numbers in 1966–2005 (9 published cases) [86].

The mean time to the appearance of oral lesions was 19.9 months, ranging from 1 to 8 years, as on average, more than 1.5 years elapsed before patients saw a specialist for control. In two cases [56,62], a specialist was even not seen until 10 to 25 years after the lesions appeared. These data suggest that the surveyed population lacks knowledge about Heck’s disease and awareness and concern about oral mucosal changes. In addition, it can be assumed that the cases of FEH probably waited an average of one and a half years after the appearance of the lesions before seeing a specialist because the lesions were asymptomatic and did not affect oral functions and esthetics [60]. Consequently, it seems necessary to make the population aware of the importance of regular oral and dental checkups, even more so in the case of oral mucosal changes. These findings can be a more comprehensive alarm signal, even if they are associated with a benign pathology, such as FEH. Indeed, oral lesions, even if asymptomatic, should never be underestimated, as they can be an early sign of other systemic diseases or malignancies. Thus, raising awareness of this issue goes hand in hand with encouraging patients to be vaccinated against HPV. 

In this regard, no study reported whether patients with manifestations of Heck’s disease received the vaccine. This may be in part because FEH is associated with low-risk HPV-13 and -32 genotypes, whereas HPV vaccines target high-risk HPV genotypes [91]. 

However, although FEH is a benign condition caused by low-risk HPV genotypes, diagnosed cases may be at higher risk for infection with other viral genotypes. Of note, coinfection with multiple HPV genotypes is recognized as a risk factor for invasive cervical cancer (ICC) and high-grade squamous intraepithelial lesions (HSILs) [92], as is HPV-HIV coinfection for various squamous cell carcinomas [93]. Therefore, awareness, particularly of pediatric patients, who are preferentially targeted by HPV vaccination, remains critical to curbing coinfection, which can further increase the risk of malignancies. 

Moreover, HPV vaccination also plays an essential role because Highly Active Antiretroviral Therapy (HAART) has been shown to be less effective for HIV–HPV coinfection-related malignancies, and to date, no vaccine against HIV is available [92]. 

Furthermore, the rate of migration from areas at a higher risk of infection to high-income countries has generally increased, proportionally increasing the number of potentially underdiagnosed cases.

Because oral FEH lesions often regress spontaneously [89] and have no cosmetic or functional consequences [84], treatment is often unnecessary [87]. However, it may be considered if the lesions become painful, interfere with occlusion, or present esthetic complications or social stigma [89,94]. Possible therapies include traditional surgery, laser surgery, electrocautery, cryotherapy, and trichloroacetic acid, excision of the lesion(s), or drug therapy with interferon, podophyllin, vitamin A, levamisole, or imiquimod [49,52,54,55,61,63,65,69,75,95]. These techniques, heterogeneously applied and associated with different success and recurrence rates, were also recorded in this systematic review [94], probably due to the lack of guidelines. 

The surgical approach of excising lesions should be limited to single and limited lesions. However, in most cases, FEH occurs as multiple nodulo-papular lesions involving large and diverse mucosa and perilabial tissue areas. 

Coherently, in this systematic review, single lesions accounted for 5.3% of cases, while the remaining 94.7% had multiple lesions, predominantly with bilateral and asymmetric patterns. In cases with greater mucosal involvement and considering the often young age of patients (first to second decade of life), conservative and atraumatic approaches may be preferred to preserve esthetics and functions. 

From this perspective, a new therapeutic approach using nano-pulse stimulation (NPS) has shown promising results [94]. This technique uses ultrashort electrical pulses to induce localized cell death [94], in contrast to cryotherapy, electrodesiccation, or laser techniques, which produce less precise and effective thermal necrosis [94]. In addition, postoperative complications were minimal [94]. However, further studies on FEH cases treated with NPS are needed to confirm the true efficacy for lesions in the oral cavity, but this technique has already shown optimal safety and efficacy in the treatment of other non-genital warts.

### 4.3. Oral Malignant HPV-Related Lesions in Pediatric Subjects: Oral Squamous Cell Carcinoma

Oral squamous cell carcinoma is a malignant epithelial neoplasm of the head and neck and ranks seventh in worldwide cancer prevalence [96]. However, it remains rare in the pediatric population [20], often associated with congenital syndromes correlated with an increased cancer risk, such as Fanconi anemia, Li-Fraumeni syndrome, Bloom syndrome, ataxia telangiectasia, and xeroderma pigmentosum [97,98], but not others associated with various oral mucosal changes. According to a recent literature review by Lee et al. [18], there were 25 reported cases of OSCC in non-syndromic patients under 16 years of age, while the review by Magalhaes et al. [1] listed a total of 42 cases of OSCC in individuals under 20 years of age in the literature since 1976.

Although its oncogenic role is still not well understood [5], HPV appears to be responsible for 3% of oral carcinomas in adults [6]. To our knowledge, two pediatric cases of OSCC [1,18] have been identified in which HPV, although the viral genotype was not reported, or HPV-positive predictive proteins have been detected. In detail, p16 positivity was detected in both cases [1,18]. The overexpression of p16 is induced by the E7 viral protein of high-risk HPV [99], such as HPV-16 and HPV-18, which are most commonly associated with OSCC [6]. Consequently, detecting high levels of p16 predicts high-risk HPV infection [5]. However, on the one hand, elevated p16 levels were also found in HPV-negative tumors, and on the other hand, many HPV-positive OSCCs were negative for p16 [4], revealing a discrepancy between HPV DNA positivity and oncogenic activity [4]. Given these considerations, although the HPV association with pediatric oral cancer has been reported based on the p16 status of the tumors, the role of HPV in oral carcinogenesis in these cases is unknown, and HPV-associated OSCC would be extremely rare in a pediatric population.

The two young subjects with HPV-related OSCC were both males, which is consistent with other studies reporting a higher prevalence of OSCC in pediatric male patients [1,18] and of HPV-OSCC in adult males (70% of cases) [100], likely related to the higher rate of oral HPV infection in males, who usually experience oral rather than anogenital infections, compared with those in females [101].

In those two cases [1,18], lesions occurred in the maxillary alveolar ridge region, similar to 12 of 42 OSCC pediatric cases with the involvement of the gingival alveolar ridge described by Magalhaes et al. [1], probably due to embryonic tissue and increased stem cell activity implicated in odontogenesis until tooth eruption [1,98].

In both studies reporting HPV-related OSCC in pediatric subjects [1,18], information about possible HPV vaccination was not reported. Nevertheless, raising awareness of HPV vaccination among children and their parents and caregivers may be of even greater importance, especially in patients exposed to high-risk HPV [102]. In fact, persistent high-risk-HPV infections are associated with more than 90% of cervical cancers and several vaginal, anal, vulvar, penile, and oropharyngeal cancers [103]. 

## 5. Conclusions

The present systematic review showed that healthy pediatric patients (≤18 years of age) with HPV-related oral lesions had a mean age of 8.46 years and were most frequently diagnosed with FEH (51%), followed by squamous cell papillomas (25%), verrucae vulgaris (13%), and condyloma acuminata (9%). The HPV association with oral squamous cell carcinomas was described in two cases based on the p16 status of the tumors, and no viral genotype was reported; thus, the role of HPV in oral carcinogenesis in these cases remains unknown, and HPV-related OSCC would be extremely rare in a pediatric population [9]. The viral genotypes detected were HPV-13 (30.61%), -6 (20.41%), -11 (16.33%), HPV-2 (12.24%), -32 (10.20%), -57 (6.12%), and -16 (4.08%). No HPV vaccination was reported in any case.

Overall, these findings and the possible independent role of HPV in oral carcinogenesis underscore the importance of HPV vaccination. The currently available vaccines (bivalent, quadrivalent, and nonavalent) are the primary means of protection against HPV infection. Since the COVID-19 pandemic abruptly halted the HPV vaccination campaign, it is advisable to revive and exceed the vaccination schedule in time for the first pandemic peak. 

Oral healthcare providers, who frequently encounter benign HPV-related lesions, should also take a leading role in this scenario, both in the early diagnosis and treatment of oral HPV-related lesions and in raising awareness of HPV vaccination among pediatric patients and their parents and caregivers.

## Figures and Tables

**Table 1:** Data extracted and collected from studies reporting pediatric cases diagnosed with oral papilloma, verruca vulgaris, or condyloma acuminata. Study characteristics: first author, year, and journal of publication; study design; the reference number; funding. Study methods: sample size (n.), mean age (y.o.), gender ratio (M/F), country, comorbidities and associated ongoing treatments, relatives with similar lesions, HPV exposure (if any), time to onset of oral lesions, type of HPV vaccine administered (if any). Oral HPV-related lesions: macroscopic and microscopic features, number (single/multiple), distribution (unilateral/bilateral, asymmetric or symmetric), location, extra-oral involvement, HPV genotype. Diagnosis: definitive diagnosis, diagnostic procedure(s) performed, therapy, progression.

Adler-Storthz K., 1986 J Oral Pathol [26] Case series No funding	Participants Sample size (n.4) Mean age (14.75 y.o.; range 12–16 y.o.) Gender ratio (2M/2F) Country: MD Comorbidities: MD Ongoing treatments: MD HPV exposure: MD Relatives with similar lesions: MD Time to oral lesion onset: MD HPV vaccine: MD Vaccine type: MD	Macroscopic features: MD Number: MD Distribution: MD Location: 2 lower lip, 2 upper lip, 1 commissure Extra-oral involvement: MD Microscopic features: papillary epithelial projections extending above the adjacent mucosal surface; rete ridges at the periphery were elongated and tended to converge centrally; the epithelial papillae showed varying degrees of acanthosis, granular cell hyperplasia, and hyperparakeratosis; large vacuolated cells with dense nuclei were scattered within the stratum spinosum and granular cell layer of papillae HPV genotype detected: 1 N/A, 3 HPV-2	Diagnosis Verruca (n.4) Diagnostic procedure(s) 4 Biopsy 4 In situ hybridization Therapy MD Progression MD
Aldhafeeri K., 2020 Cureus [27] Case report No funding	Participants Sample size (n.1) Mean age (18 y.o.) Gender ratio (1F) Country: MD Comorbidities: none Ongoing treatments: MD HPV exposure: MD Relatives with similar lesions: MD Time to oral lesion onset: 5 months HPV vaccine: MD Vaccine type: MD	Macroscopic features: a fine strand of tissue over 0.5 cm in length Number: single Distribution: unilateral Location: uvula Extra-oral involvement: MD Microscopic features: finger-like papillary projections lined by hyperplastic squamous epithelium around fibrovascular cores; focal surface parakeratosis lined by benign stratified hyperplastic squamous epithelium composed of elongated hyperchromatic nuclei with eosinophilic cytoplasm HPV genotype detected: MD	Diagnosis Papilloma (n.1) Diagnostic procedure(s) Biopsy Therapy Electrocauterization Progression Healed
Babich S.B., 2003 JADA [28] Case report No funding	Participants Sample size (n.1) Mean age (4 y.o) Gender ratio (1M) Country: MD Comorbidities: none Ongoing treatments: MD HPV exposure: suspected abuse Relatives with similar lesions: father (genital warts) Time to oral lesion onset: 3 months HPV vaccine: MD Vaccine type: MD	Macroscopic features: papular asymptomatic soft-tissue mass, 4 mm in width, blunt projections (cauliflowerlike); predominantly of the same color as the normal oral mucosa, but with a small brown area at the inferior border Number: single Distribution: unilateral Location: upper lip Extra-oral involvement: 2 genital warts with peripheral satellite lesions Microscopic features: MD HPV genotype detected: 1 HPV-6 and 11	Diagnosis Condyloma acuminata (n.1) Diagnostic procedure(s) Excisional biopsy In situ hybridization Therapy MD Progression MD
Beaudenon S., 1987 J Invest Dermatol [29] Case series No funding	Participants Sample size (n.2) Mean age (15.5 y.o.; range 14–17 y.o.) Gender ratio (2M) Country: 2 Caucasians from Denmark Comorbidities: MD Ongoing treatments: MD HPV exposure: MD Relatives with similar lesions: MD Time to oral lesion onset: MD HPV vaccine: MD Vaccine type: MD	Macroscopic features: MD Number: MD Distribution: MD Location: 1 gingiva; 1 palatoglossal arch Extra-oral involvement: 1 common wart on hand; 1 none Microscopic features: MD HPV genotype detected: 1 N\A; 1 HPV-6	Diagnosis Condyloma acuminata (n.2) Diagnostic procedure(s) 2 Biopsy 2 In situ hybridization Therapy MD Progression MD
Benyo, S. 2021 Clin Case Rep [30] Case report No funding	Participants Sample size (n.1) Mean age (13 y.o.) Gender ratio (1F) Country: MD Comorbidities: none Ongoing treatments: MD HPV exposure: MD Relatives with similar lesions: MD Time to oral lesion onset: 9 months HPV vaccine: No Vaccine type: None	Macroscopic features: multiple clusters of papilloma with one large, isolated papilloma measuring 2 × 3 cm Number: multiple Distribution: bilateral asymmetrical Location: lower lip, cheeks, commissures Extra-oral involvement: (treated) multiple palmar verrucae 2 years in advance Microscopic features: squamous lesion with papillary architecture; focal koilocytic atypia. HPV genotype detected: 1 HPV-6 and 11	Diagnosis Papilloma (n.1) Diagnostic procedure(s) Biopsy In situ Hybridization Therapy Excisional biopsy of large, isolated papilloma CO_2_ laser surgery of smaller papillomas CO_2_ laser surgery of the recurrent papillomas Progression Recurrence of the smaller papillomas after 6 and 13 months
Boj, J.R. 2007 Quintessence Int [31] Case report No funding	Participants Sample size (n.1) Mean age (12 y.o.) Gender ratio (1F) Country: MD Comorbidities: none Ongoing treatments: MD HPV exposure: MD Relatives with similar lesions: MD Time to oral lesion onset: less than 15 months HPV vaccine: MD Vaccine type: MD	Macroscopic features: small soft fragment (0.25 cm in size) of papillomatous appearance with a whitish outer surface Number: single Distribution: unilateral Location: gingiva Extra-oral involvement: MD Microscopic features: tissue covered by keratinizing stratified squamous epithelium with marked hyper- and parakeratosis; superficial epithelial cells with pyknotic nuclei and perinuclear clear spaces, indicative of HPV infection; the underlying chorion showed dilated vascular spaces and chronic inflammatory changes HPV genotype detected: MD	Diagnosis Papilloma (n.1) Diagnostic procedure(s) Biopsy Therapy Laser surgery Progression Healed after 6 months
Carneiro, T.E. 2009 J Oral Sci [32] Case series No funding	Participants Sample size (n.4) Mean age (8 y.o./range 4–14 y.o.) Gender ratio (2M/2F) Country: MD Comorbidities: MD Ongoing treatments: MD HPV exposure: MD Relatives with similar lesions: MD Time to oral lesion onset: 28.3 months (range 2–72 months) HPV vaccine: MD Vaccine type: MD	Macroscopic Features: 2 cauliflower, whitish, softened/flaccid, sessile lesions of 0.3 or 0.5 cm; 2 rounded, rosy/pink/whitish, softened/flaccid pedunculated lesions of 0.2 or 0.3 cm Number: 4 single Distribution: 4 unilateral Location: 2 lower lips, 1 tongue, 1 commissure Extra-oral involvement: MD Microscopic features: normal epithelium maturation pattern, hyperparakeratosis, discrete increase in the number of cells in the basal layer (basilar hyperplasia) of the epithelium in at least one segment, koilocyte-like cells HPV genotype detected: MD	Diagnosis Papilloma (n.4) Diagnostic procedure(s) 4 Biopsy Immunohistochemistry for p53 protein Therapy MD Progression MD
Chaitanya, P. 2018 Int J Clin Pediatr Dent [33] Case report No funding	Participants Sample size (n.1) Mean age (10 y.o) Gender ratio (1F) Country: MD Comorbidities: none Ongoing treatments: MD HPV exposure: MD Relatives with similar lesions: MD Time to oral lesion onset: 1 month HPV vaccine: MD Vaccine type: MD	Macroscopic features: pale, pink-colored, pedunculated growth with finger-like projections of soft tissue (1.5 cm) Number: single Distribution: unilateral Location: hard palate Extra-oral involvement: none Microscopic features: papillary projections of parakeratinized stratified squamous epithelium of variable thickness with localized areas showing mild basilar hyperplasia with few koilocytes and enclosing connective tissue cores fibrocellular in nature, with moderate vascularity HPV genotype detected: MD	Diagnosis Papilloma (n.1) Diagnostic procedure(s) Biopsy Therapy Excisional biopsy Progression MD
De Meneses, R.K.L. 2020 Oral Surg Oral Med Oral Pathol Oral Radiol [34] Case report No funding	Participants Sample size (n.1) Mean age (9 y.o.) Gender ratio (1M) Country: MD Comorbidities: MD Ongoing treatments: MD HPV exposure: MD Relatives with similar lesions: MD Time to oral lesion onset: 18 months HPV vaccine: MD Vaccine type: MD	Macroscopic features: verrucous nodule measuring 1 cm Number: Single Distribution: Unilateral Location: lower lip Extra-oral involvement: None Microscopic Features: hyperplasia of parakeratinized squamous epithelium, with exophytic projections exhibiting acanthosis and exocytosis; clear cytoplasm cells, suggestive of koilocytosis and subjacent fibrovascular connective tissue was also observed HPV genotype detected: MD	Diagnosis Condyloma acuminata (n.1) Diagnostic procedure(s) Biopsy Therapy Excisional biopsy Progression No recurrence after 1 year
Devi, R.S. 2014 Case Rep Dent [35] Case report No funding	Participants Sample size (n.1) Mean age (10 y.o.) Gender ratio (1M) Country: MD Comorbidities: MD Ongoing treatments: MD HPV exposure: MD Relatives with similar lesions: MD Time to oral lesion onset: 7 years HPV vaccine: MD Vaccine type: MD	Macroscopic features: small disc-like mass-pedunculated (3 × 2 cm) Number: single Distribution: unilateral Location: uvula Extra-oral involvement: MD Microscopic features: pattern of finger-like projections with a central vascular zone surrounded by stratified squamous epithelium; multiple papillary folds are hyperparakeratotic and epithelium also revealed plenty of koilocytes HPV genotype detected: MD	Diagnosis Papilloma (n.1) Diagnostic procedure(s) Biopsy Therapy Excisional biopsy Progression MD
Emmanouil, D.E. 1987 Pediatr Dent [36] Case report No funding	Participants Sample size (n.1) Mean age (5 y.o) Gender ratio (1F) Country: MD Comorbidities: impetigo and gonorrhea Ongoing treatments: antibiotics HPV exposure: suspected abuse Relatives with similar lesions: none Time to oral lesion onset: MD HPV vaccine: MD Vaccinetype: MD	Macroscopic features: 2 wart-like lesions (5 mm in diameter) with verrucous appearance and a slightly paler color than normal oral mucosa. Number: multiple Distribution: unilateral Location: lower lip Extra-oral involvement: 2 skin warts Microscopic features: mushroom-shaped soft hemorrhagic tissue; the surface was velvety and verrucous in appearance; the cut surface revealed papillary pinkish-white tissue; sections of squamous epithelium manifested papillary hyperplasia and acanthosis; many of the squamous epithelial cells showed cytoplasmic vacuolization that causes flattening of the nucleus; some of the cells contained irregular hyperchromatic nuclei; the subepithelial connective tissue showed a few mononuclear inflammatory cells; no viral inclusions were identified HPV genotype detected: MD	Diagnosis Condyloma acuminata (n.1) Diagnostic procedure(s) Vaginal coltures: (+) Neisseria lactamina, Neisseria meningitidis, Neisseria gonorrhoeae Therapy Cryosurgery for skin warts Excisional biopsy for oral warts Progression No recurrence after 6 months
Liu, N. 2013 J Craniofac Surg [19] Case report National Natural Science Foundation of China; Doctoral Program of the Ministry of Education of China	Participants Sample size (n.1) Mean age (12 y.o) Gender ratio (1M) Country: China Comorbidities: none Ongoing treatments: MD HPV exposure: MD Relatives with similar lesions: none Time to oral lesion onset: 1 month HPV vaccine: MD Vaccine type: MD	Macroscopic features: pale, slightly protruded, soft, asymptomatic papules of 1–10 mm in diameter; protruded, pink masses with the surface covered by short, blunt, fine finger-like projections resembling a cauliflower configuration Number: multiple Distribution: bilateral Location: upper lip, cheeks, retromolar area, gingiva, tongue Extra-oral involvement: none Microscopic features: exophytic papillary growths, parakeratosis, and severe acanthosis of epithelium with elongated and widened rete ridges; mild epithelial dysplasia was detected; cellular swelling, edema, and perinuclear vacuoles were observed in the upper layers of the epithelium, indicating virus infection HPV genotype detected: HPV-16	Diagnosis Papilloma (n.1) Diagnostic procedure(s) Biopsy Serological tests: (-) HIV, anti-syphils antibodies PCR analysis Therapy Oral surgery Progression No recurrence after 1 year
Misir, A.F. 2013 J Indian Soc Pedod Prev Dent [22] Case report No funding	Participants Sample size (n.1) Mean age (5 y.o.) Gender ratio (1F) Country: MD Comorbidities: none Ongoing treatments: MD HPV exposure: MD Relatives with similar lesions: MD Time to oral lesion onset: 2 months HPV vaccine: MD Vaccine type: MD	Macroscopic features: soft pink-coloured lesion approximately 0.7 cm in length Number: single Distribution: unilateral Location: hard palate Extra-oral involvement: MD Microscopic features: hyperplastic squamous epithelium and multiple finger-like projections with fibrovascular core; dense connective core with benign proliferation of stratified and squamous epithelium without cell atypia HPV genotype detected: MD	Diagnosis Papilloma (n.1) Diagnostic procedure(s) Biopsy Therapy Laser surgery Progression Healed after 1 month No recurrence after 1 year
Naghashfar, Z. 1985 J Med Virol [23] Case series No funding	Participants Sample size (n.2) Mean age (12 y.o\range 9–15 y.o.) Gender ratio (1M/1F) Country: 1 Black; 1 MD comorbidities: MD Ongoing treatments: MD HPV exposure: MD Relatives with similar lesions: MD Time to oral lesion onset: 1 MD; 1 several months HPV vaccine: MD Vaccine type: MD	Macroscopic features: MD Number: MD Distribution: MD Location: 1 gingiva, 1 lip Extra-oral involvement: 1 MD; 1 wart on forehead Microscopic features: 1 multiple papillomatous structures consisting predominantly of parakeratotic stratified squamous epithelium; occasional cells within the superficial zones displayed perinuclear vacuolization, often with nuclear pyknosis HPV genotype detected: 1 N\A; 1 HPV-6	Diagnosis Papilloma (n.2) Diagnostic procedure(s) 2 Biopsy 2 Immunoperhoxidase tests for viral antigen 2 In situ hybridization Therapy MD Progression MD
Orenuga, O.O. 2018 Niger J Clin Pract [37] No funding	Participants Sample size (n.1) Mean age (5 y.o) Gender ratio (1F) Country: MD Comorbidities: MD Ongoing treatments: MD HPV exposure: MD Relatives with similar lesions: MD Time to oral lesion onset: 3 months HPV vaccine: MD Vaccine type: MD	Macroscopic features: warty pedunculated growth of size around 10 × 7 mm, pink in color, firm in consistency Number: single Distribution: unilateral Location: hard palate Extra-oral involvement: none Microscopic features: hyperkeratinized stratified squamous epithelium with thin fibrovascular connective tissue core contained small endothelial lined vascular channels and a few chronic inflammatory cells consisting mostly of lymphocytes; koilocytes cells were also seen HPV genotype detected: MD	Diagnosis Papilloma (n.1) Diagnostic procedure(s) Biopsy Therapy Excisional biopsy Progression Recurrence after 2 years
Padayachee, A. 1994 J oral Patho Med [38] Case series S.A. Medical Research Council	Participants Sample size (n.6) Mean age (11.7 y.o./range 10–15 y.o.) Gender ratio (1M/5F) Country: MD Comorbidities: MD Ongoing treatments: MD HPV exposure: MD Relatives with similar lesions: MD Time to oral lesion onset: MD HPV vaccine: MD Vaccine type: MD	Macroscopic features: MD Number: MD Distribution: MD Location: 3 lip, 1 tongue, 1 gingiva, 1 N/D oral mucosa Extra-oral involvement: 2 warts on hands, 4 none Microscopic features: MD HPV genotype detected: 3 HPV-2 and 57; 3 N/A	Diagnosis Verruca Vulgaris (n.6) Diagnostic procedure(s) Biopsy In situ hybridization Therapy MD Progression MD
Paradisi, M. 1992 Pediatr Dermatol [39] Case report No funding	Participants Sample size (n.1) Mean age (4 y.o.) Gender ratio (1M) Country: Somalia Comorbidities: MD Ongoing treatments: MD HPV exposure: MD Relatives with similar lesions: foster relatives with genital condylomata Time to oral lesion onset: 1.5 years HPV vaccine: MD Vaccine type: MD	Macroscopic features: soft and reddish papillary lesions from 1 to 10 mm Number: multiple (approximately 60) Distribution: bilateral asymmetrical Location: cheeks, upper and lower lips, tongue Extra-oral involvement: none Microscopic features: hyperplastic epithelium with acanthosis, parakeratosis, and papillomatosis; epithelial cells with pyknotic nuclei and distinct perinuclear vacuolization (keratinocytes); slight perivascular inflammatory infiltrate and dermal papillae with largely distorted capillaries in the upper dermis; slight thickening of the stratum corneum, focal areas of parakeratosis and acantholysis HPV genotype detected: 1 HPV-6 and 11	Diagnosis Condyloma acuminata (n.1) Diagnostic procedure(s) Serological tests: normal Biopsy In situ hybridization Therapy First step: interferon alpha-2a (16 doses) Second step: podophyllin (20%) in ethanol + local interferon Third step: local podophyllin Progression Healed after the first and second steps of therapy Several months later: local recurrence of small lesions Healed after third step
Percinoto, A.C.C. 2014 BMC Res Notes [40] Case report No funding	Participants Sample size (n.1) Mean age (5 y.o.) Gender ratio (1M) Country: Caucasian Comorbidities: MD Ongoing treatments: MD HPV exposure: sexual abuse Relatives with similar lesions: MD Time to oral lesion onset: MD HPV vaccine: MD Vaccine type: MD	Macroscopic features: one lesion approximately 0.4 mm diameter with a pedicle base, and another one, with a sessile base of approximately 0.6 mm in diameter; both lesions had a firm consistency, reddish appearance, and presence of whitish areas and regions of ulceration Number: multiple (2) Distribution: unilateral Location: tongue, hard palate Extra-oral involvement: MD Microscopic features: acanthotic parakeratinized stratified squamous epithelium containing nuclei with a “raisin type” format in the upper spinous layer HPV genotype detected: MD	Diagnosis Condyloma acuminata (n.1) Diagnostic procedure(s) Biopsy Therapy MD Progression MD
Piña, A.R. 2019 Med Oral Patol Oral Cir Bucal [2] Retrospective study No funding	Participants Sample size (n.4) Mean age (6.5 y.o.\range 2–10 y.o.) Gender ratio (3M/1F) Country: MD Comorbidities: MD Ongoing treatments: MD HPV exposure: MD Relatives with similar lesions: MD Time to oral lesion onset: MD HPV vaccine: MD Vaccine type: MD	Macroscopic features: 1 nodular lesions with a slightly papillary surface Number: 2 single/2 multiple Distribution: MD Location: 2 vermilion, 1 gingiva, 1 lips Extra-oral involvement: MD Microscopic features: 1 blunt hyperplastic epithelium with papillary rounded projections showing superficial koilocytes HPV genotype detected: 2 N\A; 2 HPV-6 and 11	Diagnosis Verruca Vulgaris (n.2) Condyloma acuminata (n.2) Diagnostic procedure(s) 4 In situ hybridization Therapy MD Progression MD
Premoli-de-Percoco, G. 1993 J Oral Pathol Med [41] Case series C.D.C.H.-U.C.V.	Participants Sample size (n.7) Mean age (8.7 y.o.\range 5–16 y.o.) Gender ratio (6M/1F) Country: MD Comorbidities: MD Ongoing treatments: MD HPV exposure: 2 autoinoculation Relatives with similar lesions: MD Time to oral lesion onset: MD HPV vaccine: MD Vaccine type: MD	Macroscopic features: sessile, with a broad base, and the surfaces are tough and papillary with small clefts and pits Number: MD Distribution: MD Location: 3 hard palate, 2 upper lip, 2 commissure Extra-oral involvement: 2 finger warts Microscopic features: papillomatosis, acanthosis, and hyperkeratosis; vertical tiers of hyperkeratosis are seen overlying the subunits of papillomatous elevations and granular cells with heavy, clutched keratohyalin granules found in the valleys between the elevations; the rete ridges are elongated, characterized by their inward bending at the margin of the verruca, and point radially towards the center of the lesion; vacuolated cells, with small and basophilic nuclei surrounded by a clear halo and pale cytoplasm, are found in the upper spinous and granular cell layers in most cases (koilocyte cells) HPV genotype detected: 7 N\A	Diagnosis Verruca Vulgaris (n.7) Diagnostic procedure(s) 7 Biopsy 7 In situ hybridization Therapy MD Progression MD
Puranen, M. 1996 Am J Obstet Gynecol [42] Prospective study No funding	Participants Sample size (n.8) Mean age (N\A y.o.) Gender ratio (N\A) Country: MD Comorbidities: MD Ongoing treatments: MD HPV exposure: 8 vertical transmissions Relatives with similar lesions: 8 mothers Time to oral lesion onset: MD HPV vaccine: MD Vaccine type: MD	Macroscopic features: 2 hyperplastic plicae; 1 papillary lesion; 4 small papules (1–2 mm); fibroma (3 mm) Number: 4 single\4 multiple Distribution: MD Location: 3 palate, 1 pharyngeal arcus, 4 N\A Extra-oral involvement: MD Microscopic features: MD HPV genotype detected: 1 HPV-16; 7 MD	Diagnosis Papilloma (n.1) N\A (n.7) Diagnostic procedure(s) 8 Cytologic scrapes 1 Biopsy 1 In situ hybridization PCR analysis Therapy 1 Excisional biopsy Progression MD
Sabeena, S. 2016 Indian J Med Microbiol [43] Case report ICMR	Participants Sample size (n.1) Mean age (4 y.o) Gender ratio (1M) Country: North Kerala Comorbidities: None Ongoing treatments: MD HPV exposure: MD Relatives with similar lesions: none Time to oral lesion onset: 2 months HPV vaccine: MD Vaccine type: MD	Macroscopic features: white-colored warty lesion on the mucosal aspect Number: single Distribution: unilateral Location: lower and upper lip Extra-oral involvement: MD Microscopic features: papillomatous tumor with hyperkeratosis, acanthosis, and cells with koilocytic change HPV genotype detected: HPV-32	Diagnosis Papilloma (n.1) Diagnostic procedure(s) Biopsy PCR analysis Therapy Electrocauterization Progression Lip lesion healed after 1 month and small satellite sparse lesions were noticed
Sadaksharam, J. 2019 Indian J Med Res [44] Case report No funding	Participants Sample size (n.1) Mean age (6 y.o) Gender ratio (1M) Country: MD Comorbidities: MD Ongoing treatments: MD HPV exposure: MD Relatives with similar lesions: MD Time to oral lesion onset: MD HPV vaccine: MD Vaccine type: MD	Macroscopic features: pinkish pedunculated growths Number: multiple (3) Distribution: unilateral Location: vestibule, hard palate, tongue Extra-oral involvement: none Microscopic features: multiple exophytic finger-like projections; hyperplastic stratified squamous epithelium with hyperparakeratosis and papillomatosis enclosing connective tissue core HPV genotype detected: MD	Diagnosis Papilloma (n.1) Diagnostic procedure(s) Biopsy Therapy Excisional biopsy Progression No recurrence after 3 months
Sinclair, K.A. 2005 Pediatrics [25] Retrospective study Funding	Participants Sample size (n.17) Mean age (4.65 y.o.\range 1.5–12.7 y.o.) Gender ratio (7M/10F) Country: 9 white; 6 Black; 2 Latino Comorbidities: MD Ongoing treatments: MD HPV exposure: MD Relatives with similar lesions: MD Time to oral lesion onset: MD HPV vaccine: MD Vaccine type: MD	Macroscopic features: MD Number: MD Distribution: MD Location: MD Extra-oral involvement: 7 anogenital warts Microscopic features: MD HPV genotype detected: MD	Diagnosis Papilloma (n.17) Diagnostic procedure(s) 12 Biopsy Therapy MD Progression MD
Squires, J. 1999 Arch Pediatr Adolesc Med [45] Case series No funding	Participants Sample size (n.3) Mean age (6 y.o.\range 3–9 y.o.) Gender ratio (3F) Country: 1 Caucasian, 2 MD Comorbidities. 2 none, 1 acute respiratory illness Ongoing treatments: 1 Acyclovir HPV exposure: 2 sexual abuse Relatives with similar lesions: 1 none; 1 mother; 1 MD Time to oral lesion onset: 1 several months; 1 less than 6 months; 1 4 months HPV vaccine: MD Vaccine type: MD	Macroscopic features: 1 well circumscribed papillomatous lesion; 1 sessile lesion, generalized hyperplasia, pedunculated lesions; 1 papillomatous mass of 1 cm Number: 1 single\2 multiple Distribution: 1 bilateral Location: 2 tongue, 2 cheeks, 1 commissure, 2 hard palate Extra-oral involvement: 3 none Microscopic features: MD HPV genotype detected: MD	Diagnosis Condyloma acuminata (n.3) Diagnostic procedure(s) 3 Serological test: 3 (-) Treponema pallidum, HIV, and hepatitis B 3 Swabs 3 In situ hybridization Therapy 1 First step: 1 laser surgery; Second step: oral cimetidine for 5 months 2 First step: laser surgery; 2 Second step: laser surgery Progression 1 Recurrence after 1 weeks and worsening after 3 months since the first-step therapy and worsening 10 months since the second step therapy; healed after 1 year 1 Recurrence after 3 months since first-step therapy; no recurrence after 4 months since second step therapy 1 Recurrence after 1 month since the first step therapy and after the second step
Swan, R.H. 1981 Oral Surg Oral Med Oral Pathol [46] Case report No funding	Participants Sample size (n.1) Mean age (18 y.o) Gender ratio (1M) Country: Caucasian Comorbidities: MD Ongoing treatments: podophyllin for genital lesions HPV exposure: MD Relatives with similar lesions: MD Time to oral lesion onset: 3 months and other 2 weeks HPV vaccine: MD Vaccine type: MD	Macroscopic features: pedunculated cauliflower-like growth measuring approximately 1 cm in diameter; four smaller enlarged filiform and fungiform papillae; small, smooth-surfaced, white or pink bumps or nodules that became enlarged rapidly over a period of days to resemble cockscombs Number: multiple (13) Distribution: tendency to coalesce Location: tongue, upper lip Extra-oral involvement: genital lesions Microscopic features: papillomatosis with parakeratosis, hypergranulosis, and marked acanthosis, scattered vacuolated cells in the spinous layer and occasional mitoses in the basal region; in the superficial subepithelial connective tissue, there were numerous dilated blood vessels, and there was a mild infiltrate of lymphocytes and plasma cells HPV genotype detected: MD	Diagnosis Condyloma acuminata (n.1) Diagnostic procedure(s) Biopsy Therapy First step: podophyllin Second step: excisional biopsy Third step: excisional biopsy Fourth step: excisional biopsy Progression No improvement after first-step therapy New lesions appeared Worsening of some lesions Healing of other lesions New lesion appeared No recurrence for 6 months
Wadhera, R. 2012 EJENTAS [47] Case report	Participants Sample size (n.1) Mean age (7 y.o.) Gender ratio (1M) Country: MD Comorbidities: none Ongoing treatments: MD HPV exposure: MD Relatives with similar lesions: MD Time to oral lesion onset: 2 months HPV vaccine: MD Vaccine type: MD	Macroscopic features: pink-colored pedunculated mass measuring 3 × 2 cm with numerous finger-like projections; firm in consistency Number: single Distribution: unilateral Location: uvula Extra-oral involvement: MD Microscopic features: performed but N\A HPV genotype detected: MD	Diagnosis Papilloma (n.1) Diagnostic procedure(s) X-ray Direct laryngoscopy Therapy Excisional biopsy Progression No recurrence after 6 months
Yoshpe, N.S. 1995 J Pediatr Otorhinolaryngol [48] Case series No funding	Participants Sample size (n.2) Mean age (5 y.o.; range 3–10 y.o.) Gender ratio (1M/1F) Country: 2 Caucasian Comorbidities: MD Ongoing treatments: MD HPV exposure: 2 suspected abuse Relatives with similar lesions: MD Time to oral lesion onset: MD HPV vaccine: MD Vaccine type: MD	Macroscopic features: MD Number: 2 single Distribution: 2 unilateral Location: 2 tonsil Extra-oral involvement: isolated laryngeal papilloma Microscopic features: MD HPV genotype detected: 1 HPV-6 and 11	Diagnosis Papilloma (n.2) Diagnostic procedure(s) 1 In situ hybridization Therapy MD Progression MD

Abbreviations: number, “n.”, years old, “y.o.”; missing data, “MD”; not available “N\A”; not defined, “N\D”; human papillomavirus, “HPV”; focal epithelial hyperplasia, “FEH”; oral squamous cell carcinoma, “OSCC”; human immunodeficiency virus, “HIV”; human hepatitis B virus, “EBV”; polymerase chain reaction, “PCR”; computed tomography, “CT”; computed tomography cone beam, “CBCT”; magnetic resonance imaging, “MRI”; trichloroacetic acid, “TCA”.

**Table 2:** Extracted and collected data from studies reporting pediatric cases diagnosed with oral focal epithelial hyperplasia. Study characteristics: first author, year, and journal of publication; study design; the reference number; funding. Study methods: sample size (n.), mean age (y.o.), gender ratio (M/F), country, comorbidities and associated ongoing treatments, relatives with similar lesions, HPV exposure (if any), time to onset of oral lesions, type of HPV vaccine administered (if any). Oral HPV-related lesions: macroscopic and microscopic features, number (single/multiple), distribution (unilateral/bilateral, asymmetric or symmetric), location, extra-oral involvement, HPV genotype. Diagnosis: definitive diagnosis, diagnostic procedure(s) performed, therapy, progression.

Akyol A., 2003 Int J of Dermatol [49] Case report No funding	Participants Sample size (n.1) Mean age (17 y.o.) Gender ratio (1M) Country: MD Comorbidities: none Ongoing treatments: MD HPV exposure: MD Relatives with similar lesions: MD Time to oral lesion onset: 7 years HPV vaccine: MD Vaccine type: MD	Macroscopic features: asymptomatic papillomatous lesions Number: multiple Distribution: tendency to coalesce Location: lower lip, cheeks, gingivae, tongue Extra-oral involvement: MD Microscopic features: irregular hyperplasia of the epidermis, hypergranulosis, numerous vacuolated cells containing viral inclusion bodies in the upper layers, elongated rete ridges, and sparse perivascular lymphocyte infiltration HPV genotype detected: MD	Diagnosis FEH (n.1) Diagnostic procedure(s) Biopsy Therapy First step: levamisole, interferon, acitretin, electocauterization Second step: three sessions of CO_2_ laser surgery and interferon alpha-2b Third step: fourth session of CO_2_ laser surgery and interferon alpha-2b Progression No improvement within 3 years of first step Improvement at the end of the second step
Beaudenon, S. 1987 J Invest Dermatol [29] Case series No funding	Participants Sample size (n.1) Mean age (18 y.o.) Gender ratio (1M) Country: 1 Greenlandic Eskimos; Comorbidities: MD Ongoing treatments: MD HPV exposure: MD Relatives with similar lesions: MD Time to oral lesion onset: MD HPV vaccine: MD Vaccine type: MD	Macroscopic features: MD Number: MD Distribution: MD Location: 1 lower lip Extra-oral involvement: 1 common wart on the hand Microscopic features: MD HPV genotype detected: 1 HPV-13	Diagnosis FEH (n.1) Diagnostic procedure(s) Biopsy In situ hybridization Therapy MD Progression MD
Bennett, L.K. 2009 Pediatr Dermatol [50] Case report No funding	Participants Sample size (n.1) Mean age (9 y.o) Gender ratio (1F) Country: Hispanic Comorbidities: none Ongoing treatments: none HPV exposure: MD Relatives with similar lesions: MD Time to oral lesion onset: 1 year HPV vaccine: MD Vaccine type: MD	Macroscopic features: soft, pink, noninflammatory exophytic, flat-topeed 2 to 6 mm papules Number: multiple Distribution: MD Location: cheeks, gingiva, lip Extra-oral involvement: MD Microscopic Features: acanthotic epithelium, elongation of the rete ridges and occasional perinuclear vacuoles HPV genotype detected: HPV-13	Diagnosis FEH (n.1) Diagnostic procedure(s) Biopsy In situ hybridization Therapy None Progression MD
Binder, B. 2007 Pediatric Dermatol [51] Case report No funding	Participants Sample size (n.1) Mean age (3 y.o.) Gender ratio (1M) Country: Africa Comorbidities: none Ongoing treatments: MD HPV exposure: MD Relatives with similar lesions: MD Time to oral lesion onset: a few months HPV vaccine: MD Vaccine type: MD	Macroscopic features: soft, asymptomatic, round, flat-topped, pale papules Number: multiple Distribution: bilateral asymmetrical Location: upper and lower lips, tongue Extra-oral involvement: none Microscopic features: non-cornified, highly acanthotic epithelium with elongated rete ridges, numerous mitoses, koilocytosis with perinuclear vacuolation, and intracytoplasmic basophilic granules; the underlying connective tissue displayed elongated papillae with dilated capillaries HPV genotype detected: HPV-32	Diagnosis FEH (n.1) Diagnostic procedure(s) Biopsy Sierological tests: normal, (-) *Treponema pallidum* and HIV Therapy Topical imiquimod (refused) Progression MD
Bombeccari, G.P. 2009 JEADV [52] Case report No funding	Participants Sample size (n.1) Mean age (14 y.o.) Gender ratio (1M) Country: Ecuador Comorbidities: MD Ongoing treatments: MD HPV exposure: MD Relatives with similar lesions: none Time to oral lesion onset: MD	Macroscopic features: sessile coalescing papules Number: multiple Distribution: bilateral asymmetrical Location: lip, cheeks, tongue Extra-oral involvement: none Microscopic features: exophytic squamous epithelial proliferation with papillomatosis and mild hyperparakeratosis HPV genotype detected: HPV-13	Diagnosis FEH (n.1) Diagnostic procedure(s) Incisional biopsy PCR analysis Therapy Diode laser Progression No recurrence after 1 year
Borborema-Santos, C.M. 2006 Braz Dent J [53] Case series No funding	Participants Sample size (n.5) Mean age (N\A y.o./range 3–17 y.o.) Gender ratio (3M/2F) Country: 5 Central Amazonian Indian communities (Brazil) Comorbidities: MD Ongoing treatments: MD HPV exposure: MD Relatives with similar lesions: 2 of the 5 patients are siblings Time to oral lesion onset: MD HPV vaccine: MD Vaccine type: MD	Macroscopic features: soft papular whitish to normally mucosal lesions Number: 5 multiple Distribution: MD Location: lower and upper lip, cheeks, tongue Extra-oral involvement: MD Microscopic features: none HPV genotype detected: 5 HPV-13	Diagnosis FEH (n.5) Diagnostic procedure(s) 5 Cytobrush 5 PCR analysis Therapy MD Progression MD
Brehm, B.A. 2016 Pediatr Dermatol [54] Case report No funding	Participants Sample size (n.1) Mean age (11 y.o.) Gender ratio (1F) Country: MD Comorbidities: none Ongoing treatments: MD HPV exposure: MD Relatives with similar lesions: MD Time to oral lesion onset: 4 years HPV vaccine: MD Vaccine type: MD	Macroscopic features: pink verrucous papules of varying sizes Number: multiple Distribution: bilateral Location: upper and lower lip, tongue, cheeks, gingiva, oral floor Extra-oral involvement: none Microscopic features: hyperplastic squamous epithelium with papillomatosis, acanthosis, and parakeratosis; the rete ridges were widened, elongated, and broad; within the stratum spinosum, there were multiple cells with clumping of nuclear chromatin (mitosoid bodies) and cells with perinuclear vacuolization and irregular nuclei (koilocytes). HPV genotype detected: HPV-13	Diagnosis FEH (n.1) Diagnostic procedure(s) Blood test: normal Serological test: (-) HIV PCR analysis Therapy Laser and plastic surgery Progression Fewer smaller papules
Cohen, P.R. 1993 Pediatr Dermatol [55] Case report PHS grant DE08061	Participants Sample size (n.2) Mean age (6.5 y.o.\range 5–8 y.o.) Gender ratio (2F) Country: 2 Mexico Comorbidities: MD Ongoing treatments: MD HPV exposure: 1 suspected abuse Relatives with similar lesions: patients are sisters Time to oral lesion onset: 1 year HPV vaccine: MD Vaccine type: MD	Macroscopic features: papules of 2–4 mm Number: 2 multiple Distribution: 2 bilateral asymmetrical Location: 2 lower lips, 1 tongue Extra-oral involvement: MD Microscopic features: localized hyperplasia of the epithelium; mild parakeratosis overlaid the acanthotic epithelium, which contained occasional vacuolated cells in the upper layers and random cells demonstrating mitosis-like nuclear degeneration; in the middle and deeper layers of the epithelium, there were a few binucleated cells; the rete ridges were irregularly elongated and showed horizontal anastomosis; there were several dilated vessels in the superficial lamina propria and a sparse perivascular, predominantly lymphocytic infiltrate HPV genotype detected: MD	Diagnosis FEH (n.2) Diagnostic procedure(s) 1 Biopsy 1 In situ hybridization Therapy 1 First step: no therapy 1 Second step: topical podophyllin (25%) elixir in benzoin (5.6) 1 Third step: cryotherapy Progression Worsening of lesions in number and size No improvement after second step of therapy Recurrence of lesions after third step therapy Spontaneous improvement during the following month Healed after 1 year No recurrence after 6 months
Durso, B.C. 2005 J Cant Dent Assoc [56] Case report No funding	Participants Sample size (n.1) Mean age (21 y.o. but with oral lesions from 11 y.o.) Gender ratio (1F) Country: MD Comorbidities: MD Ongoing treatments: MD HPV exposure: MD Relatives with similar lesions: MD Time to oral lesion onset: 10 years HPV vaccine: MD Vaccine type: MD	Macroscopic features: several sessile normochromic papulonodular lesions Number: multiple Distribution: bilateral asymmetrical Location: cheek, lip, tongue Extra-oral involvement: MD Microscopic Features: “hyperkeratinized stratified epithelium exhibiting hyperplasia, acanthosis, parakeratosis and deep papillomatous projections; some squamous cells exhibited mitotic figures, called mitosoid cells; the underlying connective tissue was well supplied with collagen and well vascularized, with some congested vessels; there is a lack of inflammatory response and koilocytic cells with perinuclear halo” HPV genotype detected: MD	Diagnosis FEH (n.1) Diagnostic procedure(s) Serological tests: normal Therapy None Progression No alterations after 1 year
Falaki, F. 2009 J Oral Pathol Med [57] Case series No funding	Participants Sample size (n.6) Mean age (12.3 y.o.\range 9–15 y.o.) Gender ratio (1M/5F) Country: 6 Iran Comorbidities: MD Ongoing treatments: MD HPV exposure: MD Relatives with similar lesions: MD Time to oral lesion onset: mean of 4.2 months (range 1–8 months) HPV vaccine: MD Vaccine type: MD	Macroscopic features: soft non-tender well circumscribed flat and sessile papules with a color of surrounding mucosa Number: 6 multiple Distribution: bilateral asymmetrical and tendency to coalesce Location: 1 lip, 5 MD Extra-oral involvement: MD Microscopic features: epithelial hyperplasia with acanthosis, parakeratosis, koilocytosis, and mitosis figure HPV genotype detected: 1 N\A; 4 HPV-13; 1 HPV-32	Diagnosis FEH (n.6) Diagnostic procedure(s) Biopsy PCR analysis Therapy None Progression 4 No regression 1 Regression after 5 years 1 Regression after 2 years
Garlick, J.A. 1989 J Oral Pathol Med [58] Case series No funding	Participants Sample size (n.3) Mean age (11.7 y.o\range 6–16 y.o.) Gender ratio (3F) Country: Libyan Comorbidities: MD Ongoing treatments: MD HPV exposure: MD Relatives with similar lesions: patients 1 and 2 were sisters, patient 3 was a cousin Time to oral lesion onset: 2 MD; 6 months HPV vaccine: MD Vaccine type: MD	Macroscopic features: nodular soft, round, and circumscribed lesions of similar color to the adjacent mucosa Number: 3 multiple Distribution: bilateral asymmetrical Location: 3 upper and 3 lower lips, 3 cheeks, 3 commissure, 3 vermillion Extra-oral involvement: 3 skin warts adjacent lips, 1 hands wart Microscopic features: (n.3) “hyperplasia of the epithelium typified by mild hyperkeratosis, acanthosis, and elongation and anastomosis of rete ridges; (n.1) condensation of keratohyalin granules in the spinous layer, yet showed very few mitosoid cells or koilocytes; (n.1) numerous koilocytes and occasional mitosoid cells in the lower stratum spinosum; (n.1) numerous swollen cells with a pale cytoplasm that demonstrated a ballooning type of degeneration, many of these cells showed pyknotic nuclei and a vacuolated cytoplasm, occasional mitosoid cells, and condensation of keratohyaline granules were also seen in the spinous layer” HPV genotype detected: 3 HPV-13	Diagnosis FEH (n.3) Diagnostic procedure(s) 3 Biopsy Therapy MD Progression MD
Hall, C. 2010 Oral Surg Oral Med Oral Pathol Oral Radiol Endod [59] Case report No funding	Participants Sample size (n.3) Mean age (8.3 y.o.\range 5–13 y.o.) Gender ratio (1M/2F) Country: 3 Australia Comorbidities: MD Ongoing treatments: MD HPV exposure: MD Relatives with similar lesions: three patients were siblings Time to oral lesion onset: MD HPV vaccine: MD Vaccine type: MD	Macroscopic features: exophytic, sessile, smooth-surfaced nodules ranged in size from 1 to 3 mm in diameter, firm on palpation, and covered by normal pink mucosa Number: 3 multiple Distribution: 3 bilateral asymmetrical Location: 3 tongue, 2 lower lip Extra-oral involvement: MD Microscopic features: 1 polypoid lesion consisting of a fibrous core covered by hyperplastic squamous epithelium with occasional dyskeratotic and mitosoid cells HPV genotype detected: MD	Diagnosis FEH (n.3) Diagnostic procedure(s) 1 Incisional biopsy Therapy MD Progression MD
Hashemipour, M.A. 2010 Arch Iran Med [18] Case report No funding	Participants Sample size (n.1) Mean age (12 y.o.) Gender ratio (1F) Country: MD Comorbidities: MD Ongoing treatments: MD HPV exposure: MD Relatives with similar lesions: MD Time to oral lesion onset: 8 years HPV Vaccine: MD Vaccine type: MD	Macroscopic features: lesions with a wide range of sizes, from 5 to 10 mm of the same color as mucosa with no inflammatory appearance, the surface was soft with firm consistency and pediculated Number: multiple Distribution: bilateral Location: vestibule, lower lip, cheek Extra-oral involvement: MD Microscopic features: squamous epithelium with prominent acanthosis and broad elongated rete ridges, epithelial dysplasia was not detected, but there were very isolated mitosoid cells HPV genotype detected: MD	Diagnosis FEH (n.1) Diagnostic procedure(s) Serological tests: normal Incisional biopsy PCR analysis Therapy CO_2_ laser surgery Progression Healed after 2 weeks
Liu, N. 2012 Int J Oral Maxillofac Surg [60] Case series National Natural Science Foundation of China; Science Funds for Talented Professionals of Sichuan Province in China	Participants Sample size (n.1) Mean age (7 y.o.) Gender ratio (1F) Country: China Comorbidities: none Ongoing treatments: MD HPV exposure: MD Relatives with similar lesions: none Time to oral lesion onset: 1 years HPV vaccine: MD Vaccine type: MD	Macroscopic features: slightly protruded, soft papules with the same color as the surrounding mucosa or wither, ranging from 3 to 10 mm in diameter Number: multiple Distribution: bilateral Location: lips, cheeks Extra-oral involvement: none Microscopic features: parakeratosis and severe acanthosis with elongated and widened rete ridges; epithelial dysplasia was not detected; the nuclei of epithelial cells in the stratum spinosum were enlarged and hyperchromatic with very little nuclear degeneration resembling a mitotic figure (mitosoid cell) HPV genotype detected: HPV-13	Diagnosis FEH (n.1) Diagnostic procedure(s) Serological tests: (-) HIV and antisyphilis antibodies Biopsy PCR analysis Therapy None Progression MD
Lorduy, C.M. 2018 Gen Dent [21] Prospective study No funding	Participants Sample size (n.10) Mean age (MD/range 0–10 y.o.) Gender ratio (N\A) Country: MD Comorbidities: no allergy Ongoing treatments: none HPV exposure: MD Relatives with similar lesions: MD Time to oral lesion onset: MD HPV vaccine: MD Vaccine type: MD	Macroscopic features: MD Number: MD Distribution: MD Location: MD Extra-oral involvement: MD Microscopic features: MD HPV genotype detected: MD	Diagnosis FEH (n.10) Diagnostic procedure(s) MD Therapy 10 TCA (5–7 applications) Progression 10 No recurrence after 1 years
Lutzner, M. 1982 Arch Dermatol [61] Case series Institut National de la Santé et de la Recherche Médicale	Participants Sample size (n.2) Mean age (8 y.o.\range 6–19 y.o.) Gender ratio (1M/1F) Country: 1 Algerian, 1 Moroccan Comorbidities: MD Ongoing treatments: MD HPV exposure: MD Relatives with similar lesions: two brothers and one sister Time to oral lesion onset: 5 months; 1 year HPV vaccine: MD Vaccine type: MD	Macroscopic features: soft, slightly elevated, oval-to-round papules, 2 to 5 mm in diameter, with a pink or white surface Number: multiple Distribution: bilateral/tendency to coalesce Location: 2 lips, 2 cheeks, 1 gingiva, 1 tongue, 1 hard palates Extra-oral involvement: 2 skin warts; 1 wart on hands Microscopic features: excised lesions from patients 1 and 2 showed acanthosis of a peculiar type exhibiting irregular elongation, lateral anastomoses, and horizontal, interconnecting branching of the rete ridges (so-called bronze-age ax sign); a few cells throughout the mucosa contained nuclei with abnormal dense granules that in some instances, mimicked mitotic figures; this appearance has been called “mitosoid” degeneration; other cells, especially in the outermost layer, were binuclear; some mononuclear and some binuclear cells exhibited a perinuclear clear zone; in lesions from both patients, a small number of papillomavirus-like particles could be seen in some cells of the outer layer of mucosa to be dispersed sparsely throughout the nucleus; a cell exhibiting a mitosoid nucleus was seen, and the dense, nuclear bodies appeared to be composed of condensed chromatin-like material; since this cell possessed a nuclear membrane, it is unlikely that it was in metaphase; some binuclear cells were infected with papillomavirus-like particles in both nuclei HPV genotype detected: MD	Diagnosis FEH (n.2) Diagnostic procedure(s) Biopsy Therapy 2 Shaving and electrocoagulation Progression 1 No recurrence after 1.5 months
Mansouri, Z. 2015 Iran J Pathol [62] Case report No funding	Participants Sample size (n.1) Mean age (35 y.o. but with oral lesions from 15 y.o.) Gender ratio (1M) Country: Iran Comorbidities: none Ongoing treatments: MD HPV exposure: MD Relatives with similar lesions: MD Time to oral lesion onset: 25 years HPV vaccine: MD Vaccine type: MD	Macroscopic features: soft, sessile, smooth surface papules and nodules ranged from 2–10 mm in diameter Number: multiple\tendency to coalesce Distribution: bilateral Location: upper and lower lips, cheeks Extra-oral involvement: MD Microscopic features: squamous epithelium with focal parakeratosis, hyperkeratosis, acanthosis, verrucous proliferation, and marked papillomatosis; hyperplasia of basal cells, and isolated perinuclear cellular vacuolization (koilocytosis), cellular binucleation, and nuclear irregularities were other features; there were well isolated mitosoid cells, but not dysplasia HPV genotype detected: MD	Diagnosis FEH (n.1) Diagnostic procedure(s) Biopsy Therapy None Progression MD
Martins, W.D. 2006 Int J Paediatr Dent [63] Case report No funding	Participants Sample size (n.1) Mean age (14 y.o.) Gender ratio (1F) Country: white Brazialian Comorbidities: MD Ongoing treatments: MD HPV exposure: MD Relatives with similar lesions: none Time to oral lesion onset: several years HPV vaccine: MD Vaccine type: MD	Macroscopic features: elevated sessile, smooth-surfaced nodules firm on palpation, covered by healthy normal-appearing mucosa, ranging in size from 1 to 3 mm in diameter Number: multiple (4) Distribution: MD Location: gingiva Extra-oral involvement: MD Microscopic features: epithelial hyperplasia with acanthosis and hydropic degeneration, parakeratosis, some mitosoidal cells; the rete ridges were frequently joined (the so-called “Bronze Age battle-axe” or “clubs” appearance) HPV genotype detected: HPV-13	Diagnosis FEH (n.1) Diagnostic procedure(s) Biopsy PCR analysis Therapy Excisional biopsy Progression No recurrence after over 1 year
Moussavi, S. 1986 J Am Dent Assoc [64] Case series No funding	Participants Sample size (n.2) Mean age (9.5 y.o.\range 6–13 y.o.) Gender ratio (2F) Country: 2 Iran Comorbidities: MD Ongoing treatments: MD HPV exposure: MD Parents with similar lesions: MD Time to oral lesion onset: 1 MD; 1 two years HPV vaccine: MD Vaccine type: MD	Macroscopic features: small nodular and papular lesions Number: 2 multiple Distribution: 1 unilateral/1 bilateral Location: 1 upper and 1 lower lip, 1 cheek Extra-oral involvement: MD Microscopic features: acanthosis and papillomatosis of the stratified squamous epithelium with elongation and anastomosing of the rete ridges; vacuolar degeneration of the spinous layer; the surface layer was slightly parakeratotic; a mild chronic inflammatory cell infiltrate was noticed in the underlying lamina propria HPV genotype detected: MD	Diagnosis FEH (n.2) Diagnostic procedure(s) 2 Biopsy Therapy MD Progression 1 Recurrence after 6 months; improvement after 3 months later; 1 healed after 2 months later 1 Improvement after 4 months
Nallanchakrava, S. 2018 Int J Clin Pediatr Dent [65] Case report No funding	Participants Sample size (n.1) Mean age (5 y.o.) Gender ratio (1M) Country: MD Comorbidities: none Ongoing treatments: MD HPV exposure: MD Relatives with similar lesions: MD Time to oral lesion onset: 3 months HPV vaccine: MD Vaccine type: MD	Macroscopic features: soft, sessile papules varying 2–10 cm in dimension Number: multiple (3) Distribution: Bilateral asymmetrical Location: lower lip, tongue Extra-oral involvement: none Microscopic features: benign parakeratotic hyperplastic mucosa with marked papillomatosis and acanthosis, and some of the cells showed isolated perinuclear vacuolization and the presence of occasional mitosoid cells; there was no evidence of dysplasia HPV genotype detected: HPV-32	Diagnosis FEH (n.1) Diagnostic procedure(s) Blood tests: normal Biopsy PCR analysis Therapy Laser surgery Progression Healed after 1 month
Nartey, N.O. 2002 J Clin Pediatr Dent [66] Case series No funding	Participants Sample size (n.6) Mean age (8.7 y.o.\range 4–12 y.o.) Gender ratio (1M/5F) Country: Ghana Comorbidities: MD Ongoing treatments: MD HPV exposure: MD Relatives with similar lesions: none Time to oral lesions onset: MD HPV vaccine: MD Vaccine type: MD	Macroscopic features: papulonodular or papillomatous type of lesions with sessile bases Number: 6 multiple Distribution: MD Location: 5 lips, 5 cheeks, 3 commissure, 2 gingiva, 1 oral floor, 1 tongue Extra-oral involvement: MD Microscopic features: fibroconnective tissue surfaced by parakeratinised stratified squamous epithelium characterized by acanthosis, bulbous rete ridges, and anastomoses of the rete ridges HPV genotype detected: MD	Diagnosis FEH (n.6) Diagnostic procedure(s) Biopsy Therapy None Progression 4 Spontaneous regression after 18 months 2 Spontaneous regression after 3 years
Ozden, B. 2011 J Maxillofac Oral Surg [67] Case report No funding	Participants Sample size (n.1) Mean age (7 y.o.) Gender ratio (1F) Country: Caucasian Comorbidities: none Ongoing treatments: MD HPV exposure: MD Relatives with similar lesions: MD Time to oral lesion onset: 6 months HPV vaccine: MD Vaccine type: MD	Macroscopic features: soft, non-ulcerated and not inflamed sessile papules and nodules from 2 to 10 mm Number: multiple Distribution: MD Location: upper lip, cheeks Extra-oral involvement: none Microscopic features: squamous epithelium with focal parakeratosis, hyperkeratosis, acanthosis, verrucous proliferation, and marked papillomatosis, hyperplasia of basal cells, isolated perinuclear cellular vacuolization (koilocytosis), cellular binucleation, and nuclear irregularities; the presence of epithelial dysplasia was not detected; there were well-isolated mitosoid cells HPV genotype detected: HPV-32	Diagnosis FEH (n.1) Diagnostic procedure(s) Skin test: normal Serological tests: normal Biopsy PCR analysis Therapy None Progression No improvement after 18 months
Pfister, H. 1983 J Virol [68] Case report Deutsche Forschungsgemeinschaft	Participants Sample size (n.1) Mean age (13 y.o) Gender ratio (1F) Country: Turkey Comorbidities: MD Ongoing treatments: MD HPV exposure: MD Relatives with similar lesions: MD Time to oral lesion onset: 3 years HPV vaccine: MD Vaccine type: MD	Macroscopic features: plaques Number: multiple Distribution: MD Location: upper and lower lips, commissures, cheeks Extra-oral involvement: skin warts Microscopic features: strong acanthosis with elongated rete ridges, a significant papillomatosis, and a continuous parakeratosis; large vacuolated cells with deeply basophilic nuclei were found focally in both the lower and the upper stratum malpighii HPV genotype detected: HPV-13	Diagnosis FEH (n.1) Diagnostic procedure(s) In situ hybridization Therapy MD Progression MD
Piña, A.R. 2019 Med Oral Patol Oral Cir Bucal [2] Retrospective study No funding	Participants Sample size (n.1) Mean age (11 y.o.) Gender ratio (1M) Country: MD Comorbidities: MD Ongoing treatments: MD HPV exposure: MD Relatives with similar lesions: MD Time to oral lesion onset: MD HPV vaccine: MD Vaccine type: MD	Macroscopic features: MD Number: 1 multiple Distribution: MD Location: 1 lips Extra-oral involvement: MD Microscopic features: MD. HPV genotype detected: 1 HPV-6 and 11	Diagnosis FEH (n.1) Diagnostic procedure(s) 1 In situ hybridization Therapy MD Progression MD
Premoli-de-Percoco, G. 1992 Virchows Arch A Pathol Anat Histopathol [24] Case series Concejo Desarrollo Cientifico y Humanistico—Universidad central de Venezuela; Concejo Nacional de Investigaciones Cientificas y Tecnologicas; Fundacion Polar Venezuela	Participants Sample size (n.9) Mean age (10.2 y.o.\range 6–15 y.o.) Gender ratio (3M/6F) Country: 9 Venezuela Comorbidities: MD Ongoing treatments: MD HPV exposure: MD Relatives with similar lesions: four patients are relatives; 9 patients Time to oral lesion onset: MD HPV vaccine: MD Vaccine type: MD	Macroscopic features: nodular elevations Number: 9 multiple Distribution: MD Location: 1 upper lip, 1 tongue, 8 MD Extra-oral involvement: MD Microscopic features: epithelial acanthosis, prominent clubbing and anastomosis of epithelial ridges with basal orientation, mild hyperparakeratosis, enlarged ballooning cells with abnormal nuclear chromatin patterns and multinucleated cells, cells immediately beneath the surface often displayed large cytoplasmic vacuolization with deeply basophilic nuclei HPV genotype detected: 4 HPV-13	Diagnosis FEH (n.9) Diagnostic procedure(s) 9 Biopsy 9 In situ hybridization Therapy MD Progression MD
Puriene, A. 2011 Stomatologija [69] Case report No funding	Participants Sample size (n.1) Mean age (15 y.o.) Gender ratio (1F) Country: MD Comorbidities: MD Ongoing treatments: MD HPV exposure: MD Relatives with similar lesions: MD Time to oral lesion onset: 2 years HPV vaccine: MD Vaccine type: MD	Macroscopic features: multiple slightly elevated normochromic papulonodular lesions Number: multiple Distribution: unilateral Location: cheeks, lip Extra-oral involvement: MD Microscopic features: hyperkeratinized epithelium exhibiting hyperplasia and deep papillomatous projections; acanthosis and parakeratosis are consistent findings; some squamous cells exhibited mitotic figures (koilocytic cells) HPV genotype detected: MD	Diagnosis FEH (n.1) Diagnostic procedure(s) Sexually transmitted diseases tests: negative Biopsy Therapy Excision biopsy Progression MD
Sarraj, A. 2013 Ann Stomatol [70] Case report No funding	Participants Sample size (n.1) Mean age (13 y.o.) Gender ratio (1F) Country: Hispanic Comorbidities: none Ongoing treatments: MD HPV exposure: MD Relatives with similar lesions: MD Time to oral lesion onset: MD HPV vaccine: MD Vaccine type: MD	Macroscopic features: soft, non-tender, flattened lesions of the same color of the oral mucosa Number: multiple Distribution: bilateral Location: cheeks, lip, vestibule Extra-oral involvement: none Microscopic features: acanthosis and superficial keratinocytes with koilocytic changes HPV genotype detected: MD	Diagnosis FEH (n.1) Diagnostic procedure(s) Biopsy Therapy Quantum Molecular Resonance Scalpel (3 sessions) Progression Healed
Saunders, N.R. 2010 Pediatr Infect Dis J [71] Case series No funding	Participants Sample size (n.2) Mean age (9.5 y.o.\range 8–11 y.o.) Gender ratio (1M/1F) Country: 2 southern Guyana Comorbidities: 2 none Ongoing treatments: MD HPV exposure: 1 horizontal transmission (common toothbrush); 1 N\A Relatives with similar lesions: 1 mother and sister Time to oral lesion onset: 1 several years HPV vaccine: MD Vaccine type: MD	Macroscopic features: 2 raised, well circumscribed, soft papulonodular lesions with smooth surface; ranged in color from white to normal mucosal color Number: 2 multiple Distribution: 2 unilateral, 2 bilateral, 1 tendency to coalesce Location: 2 cheeks, 2 tongue Extra-oral involvement: MD Microscopic features: MD HPV genotype detected: 2 HPV-13	Diagnosis FEH (n.2) Diagnostic procedure(s) 2 PCR analysis Therapy MD Progression MD
Starink, T.M. 1977 Br J Dermatol [72] Case series No funding	Participants Sample size (n.2) Mean age (6.5 y.o.\range 4–9 y.o.) Gender ratio (1M/1F) Country: 2 Netherlands (two Africans) Comorbidities: none Ongoing treatments: MD HPV exposure: MD Relatives with similar lesions: patients are siblings Time to oral lesion onset: 2 and 10 months HPV vaccine: MD Vaccine type: MD	Macroscopic features: 1 well-defined, slightly elevated round and ovoid, partly confluent, soft papules varying from 1 to 10 mm in diameter, normal pink or whitish with a flat to slightly verrucous surface; 1 papule measuring no more than 4 mm in diameter Number: 2 multiple Distribution: MD Location: 2 lower and 2 upper lips, 2 commissures, 1 gingiva, 2 cheeks, 2 palate, 1 tongue, 1 oral floor, 1 anterior faucial pillars Extra-oral involvement: MD Microscopic features: acanthosis and papillomatosis with elongated anastomosing rete pegs; surface layer was slightly parakeratotic; in some places nuclear degeneration and swelling of cells were present in the upper part of the stratum Malpighi; deposits of PAS-positive material Direct and indirect immunofluorescence studies: normal Electron microscopy: cells with large intracytoplasmic vacuoles and with only fragments of tonofilaments especially in the upper part of Malpighian layer; some cells show loss of cytoplasm from the center of the cell to varying degrees, intracytoplasmic organelles are scarce; mitochondria are mostly intact; the vacuoles are often located around the nucleus, the nuclear membrane is intact and nucleoli are present; some nuclei show peripheral clumping of chromatin, other nuclei show indentation of their surface, also in cells without distinct degenerative changes; some cells contain intracellular glycogen HPV genotype detected: MD	Diagnosis FEH (n.2) Diagnostic procedure(s) 1 Blood test: normal 1 Biopsy 1 Tissue culture: negative Therapy 1 Vitamin A (0.05%) Progression 1 Healed spontaneously
Tan, A.K. 1995 Otolaryngol Head Neck Surg [73] Case series No funding	Participants Sample size (n.2) Mean age (4.5 y.o.\range 4–5 y.o.) Gender ratio (2F) Country: 2 Ikalouit Comorbidities: 2 none Ongoing treatments: 2 none HPV exposure: MD Relatives with similar lesions: patients are sisters; father Time to oral lesion onset: 2 two years HPV vaccine: MD Vaccine type: MD	Macroscopic features: sharply defined, round-to-ovoid, smooth papules measuring 2 to 5 mm with color identical to that of the surrounding mucosa Number: 2 multiple Distribution: 2 bilateral Location: 2 tongue, 2 cheek Extra-oral involvement: 2 none Microscopic features: acanthosis and elongation with prominent clubbing and lateral anastomoses of the rete ridges of the oral epithelium, prominent vacuolization of cells in the upper portion of the epithelium and binucleated epithelial cells in the middle layer HPV genotype detected: MD	Diagnosis FEH (n.2) Diagnostic procedure(s) 1 Biopsy Therapy MD Progression MD
Wallace, J.R. 1976 J Am Dent Assoc [74] Case report No funding	Participants Sample size (n.1) Mean age (16 y.o.) Gender ratio (1F) Country: Black Comorbidities: MD Ongoing treatments: MD HPV exposure: MD Relatives with similar lesions: none Time to oral lesion onset: 1 year HPV vaccine: MD Vaccine type: MD	Macroscopic features: soft, raised, sessile, flat papules varying in size from 2 mm to 1 cm or larger Number: multiple Distribution: MD Location: cheeks, retromolar areas, lips Extra-oral involvement: MD Microscopic features: plaque-like elevation of the surface epithelium caused by a pronounced hyperplasia of the epithelium, broad irregular acanthosis, and club-like rete ridges; the epithelium was basophilic with prominent nuclei extending into the midportion; vacuolation was noted in the stratum Malpighi; mitotic activity was brisk in the basal portion HPV genotype detected: MD	Diagnosis FEH (n.1) Diagnostic procedure(s) Biopsy Therapy MD Progression MD
Yasar S., 2009 Pediatr Dermatol [75] Case series No funding	Participants Sample size (n.3) Mean age (8.3 y.o.; range 5–17 y.o.) Gender ratio (3F) Country: MD Comorbidities: 3 none Ongoing treatments: MD HPV exposure: MD Relatives with similar lesions: 1 MD; 2 none Time to oral lesion onset: 1 year, 3 and 6 months HPV vaccine: MD Vaccine type: MD	Macroscopic features: soft papulo-nodules (0.2–0.5 cm) of the same color as the normal oral mucosa with lobulated and verrucous surface; some were aggregated closely to form slightly elevated plaques Number: multiple (approximately 40–20–10) Distribution: 3 bilateral asymmetrical\1 tendency to coalesce Location: 2 hard palate, 1 cheeks, 3 upper and 3 lower lips Extra-oral involvement: MD Microscopic features: MD HPV genotype detected: MD	Diagnosis FEH (n.3) Diagnostic procedure(s) 3 Blood test: normal Urine test: normal 2 Serological tests: normal Therapy Levamisole 2 Imiquimod cream (5%) 3 TCA (80%) 2 Cryosurgery Progression 1 TCA + cryotherapy: no improvement; lesions resolved after imiquimod 1 TCA for 4 sessions: no improvement; lesions resolved after imiquimod 2 Herpes labialis attack during treatment 3 No recurrence after 1 year

Abbreviations: number, “n.”, years old, “y.o.”; missing data, “MD”; not available “N\A”; not defined, “N\D”; human papillomavirus, “HPV”; focal epithelial hyperplasia, “FEH”; oral squamous cell carcinoma, “OSCC”; human immunodeficiency virus, “HIV”; human hepatitis B virus, “EBV”; polymerase chain reaction, “PCR”; computed tomography, “CT”; computed tomography cone beam, “CBCT”; magnetic resonance imaging, “MRI”; trichloroacetic acid, “TCA”.

**Table 3:** Extracted and collected data from studies reporting pediatric cases diagnosed with oral squamous cell carcinoma. Study characteristics: first author, year, and journal of publication; study design; the reference number; funding. Study methods: sample size (n.), mean age (y.o.), gender ratio (M/F), country, comorbidities and associated ongoing treatments, relatives with similar lesions, HPV exposure (if any), time to onset of oral lesions, type of HPV vaccine administered (if any). Oral HPV-related lesions: macroscopic and microscopic features, number (single/multiple), distribution (unilateral/bilateral, asymmetric or symmetric), location, extra-oral involvement, HPV genotype. Diagnosis: definitive diagnosis, diagnostic procedure(s) performed, therapy, progression.

Lee, N.V. 2020 Oral Surg Oral Med Oral Pathol Oral Radiol [18] Case report Dr. Michele Williams Education and Research Fund, BC Cancer Foundation	Participants Sample size (n.1) Mean age (5 y.o.) Gender ratio (1M) Country: Caucasian Comorbidities: none Ongoing treatments: MD HPV exposure: suspected autoinoculation Relatives with similar lesions: two siblings Time to oral lesion onset: MD HPV vaccine: MD Vaccine type: MD	Macroscopic features: sudden onset swelling; swelling with some whitish exudate Number: single Distribution: unilateral Location: maxillary alveolar ridge Extra-oral involvement: warts on hands, chin, philtrum, and commissures Microscopic features: sheets of proliferative squamous epithelium and occasional keratin formation, with bland cytology and absence of atypia or cellular pleomorphism; anastomosing channels lined by squamous epithelium with hyperkeratosis and microcystic structures resembling “rabbit burrows” and areas with prominent nuclear pleomorphism, hyperchromasia, and numerous atypical mitotic figures, as well as diffuse p16 positivity HPV genotype detected: N\A but positivity for p16	Diagnosis OSCC (n.1) Diagnostic procedure(s) Periapical radiograph: radiolucency apical to #E and a mesiodens between the 2 permanent central incisors CBCT: palatal soft tissues swelling and impacted mesiodens Biopsy PCR analysis Therapy First step: antibiotics Second step: extraction of #D and #E Third step: biopsy Fourth step: excisional biopsy Fifth step: marginal resection of the anterior emi-maxilla with removal of #8, #7, #6 and #C Progression No response after the first step of therapy Worsening after the second step Recurrence after the third step Healed after 4 weeks since the fifth step
Magalhaes, M.A. 2016 Oral Surg Oral Med Oral Pathol Oral Radiol [1] Case report No funding	Participants Sample size (n.1) Mean age (8 y.o.) Gender ratio (1M) Country: MD Comorbidities: none Ongoing treatments: MD HPV exposure: MD Relatives with similar lesions: MD Time to oral lesion onset: MD HPV vaccine: MD Vaccine type: MD	Macroscopic features: pink-red, oval soft-tissue mass, measuring 4 cm in maximum diameter Number: single Distribution: unilateral Location: maxillary alveolar ridge Extra-oral involvement: MD Microscopic features: “ulcerated mucosal surface; most of the tumor consisted of sheets of basaloid cells with focal areas of abrupt squamous differentiation and some of the tumor islands had a ragged periphery, with small cords and single tumor cells invading the connective tissue stroma; the basaloid cells had oval, vesicular nuclei without marked pleomorphism and numerous mitotic figures; the surface mucosa showed a focal area of dysplasia; nests of basaloid epithelium with focal squamous differentiation and focal atypia” HPV genotype detected: N\A but positivity for p16	Diagnosis OSCC (n.1) in T3N0M0 stage Diagnostic procedure(s) Biopsy CBTC: osteolytic lesion with destruction of the buccal cortex; the floor of maxillary sinus was displaced superiorly and there was a thin smooth layer of periosteal new bone over the tumor mass; the roots of the first molar were medially displaced, but there was no evidence of root resorption Incisional biopsy CT: tumor mass displacing the facial artery and the buccal fat plane laterally; the bony crypts of the developing second premolar and the second molar were mostly intact, except for defects adjacent to the lesion, and the follicular spaces of these teeth appeared uniform MRI: no evidence of disease in the regional lymph nodes In situ hybridization for HBV: negative Therapy Partial maxillectomy Progression Healed after 24 months

Abbreviations: number, “n.”, years old, “y.o.”; missing data, “MD”; not available “N\A”; not defined, “N\D”; human papillomavirus, “HPV”; focal epithelial hyperplasia, “FEH”; oral squamous cell carcinoma, “OSCC”; human immunodeficiency virus, “HIV”; human hepatitis B virus, “EBV”; polymerase chain reaction, “PCR”; computed tomography, “CT”; computed tomography cone beam, “CBCT”; magnetic resonance imaging, “MRI”; trichloroacetic acid, “TCA”.

**Table 4:** Risk of bias of all studies included in the systematic review and listed in alphabetical order. For each of the seven domains assessed: Y = low risk of bias, PY = moderate risk of bias, PN = serious risk, N = critical risk of bias, and NI = no information.

Study	Confounding	Selection of Participants	Classification of Interventions	Deviations from Intended Interventions	Bias Due to Missing Data	Measurement of Outcomes	Selection of Reported Result
Adler-Storthz et al., 1985 [26]	Y	Y	Y	Y	Y	PY	Y
Akyol et al., 2003 [49]	Y	Y	Y	PY	Y	PN	Y
Aldhafeeri et al., 2020 [27]	Y	Y	Y	Y	PY	Y	Y
Babich et al., 2003 [28]	Y	Y	Y	Y	Y	Y	Y
Beaudenon et al., 1987 [29]	Y	Y	Y	Y	Y	Y	Y
Bennett et al., 2009 [50]	Y	Y	Y	Y	PY	Y	Y
Benyo et al., 2021 [30]	Y	Y	Y	Y	PY	PN	Y
Binder et al., 2007 [51]	Y	Y	Y	Y	Y	Y	Y
Boj et al., 2007 [31]	Y	Y	Y	Y	Y	PN	Y
Bombeccari et al., 2009 [52]	Y	Y	Y	Y	Y	Y	Y
Borborema-Santos et al., 2006 [53]	Y	Y	Y	Y	Y	Y	Y
Brehm et al., 2016 [54]	Y	Y	Y	Y	PY	Y	Y
Carneiro et al., 2009 [32]	Y	Y	Y	Y	Y	Y	Y
Chaitanya et al., 2018 [33]	Y	Y	Y	Y	Y	Y	Y
Cohen et al., 1993 [55]	Y	Y	Y	Y	Y	Y	PY
De Meneses et al., 2020 [34]	Y	Y	Y	Y	Y	Y	Y
Devi et al., 2014 [35]	Y	Y	Y	Y	Y	Y	Y
Durso et al., 2005 [56]	Y	Y	Y	Y	Y	Y	Y
Emmanouil et al., 1987 [36]	PY	PY	PY	Y	PY	PN	Y
Falaki et al., 2009 [57]	Y	Y	Y	PY	PY	Y	Y
Garlick et al., 1989 [58]	Y	Y	Y	Y	Y	Y	Y
Hall et al., 2010 [59]	Y	Y	Y	Y	Y	PY	Y
Hashemipour et al., 2010 [18]	Y	Y	Y	Y	Y	Y	Y
Lee et al., 2020 [20]	PY	Y	PN	PY	PN	N	Y
Liu et al., 2012 [60]	Y	Y	Y	Y	Y	Y	Y
Liu et al., 2013 [19]	Y	Y	Y	Y	Y	Y	Y
Lorduy et al., 2018 [21]	PY	PY	Y	Y	Y	Y	Y
Lutzner et al., 1982 [61]	Y	Y	Y	Y	PY	Y	Y
Magalhaes et al., 2016 [1]	PY	PY	Y	Y	Y	PN	Y
Mansouri et al., 2015 [62]	Y	Y	Y	Y	Y	Y	Y
Martins et al., 2006 [63]	Y	Y	Y	Y	Y	Y	Y
Misir et al., 2013 [22]	Y	Y	Y	Y	Y	Y	PY
Moussavi et al., 1986 [64]	Y	Y	Y	Y	Y	Y	Y
Naghashfar et al., 1985 [23]	Y	PN	Y	PY	Y	Y	Y
Nallanchakrava et al., 2018 [65]	Y	Y	Y	Y	Y	Y	Y
Nartey et al., 2002 [66]	Y	Y	Y	Y	Y	Y	Y
Orenuga et al., 2018 [37]	Y	Y	Y	Y	Y	Y	Y
Ozden et al., 2011 [67]	Y	Y	Y	PY	PY	Y	Y
Padayachee et al., 1994 [38]	Y	PY	Y	Y	Y	Y	Y
Paradisi et al., 1992 [39]	Y	Y	Y	PY	Y	Y	PY
Percinoto et al., 2014 [40]	Y	Y	Y	Y	Y	Y	Y
Pfister et al., 1983 [68]	Y	Y	Y	Y	Y	Y	Y
Pina et al., 2019 [2]	Y	Y	Y	Y	Y	Y	Y
Premoli-de-Percoco et al., 1993 [41]	Y	Y	Y	Y	Y	Y	Y
Premoli-de-Percoco et al., 1992 [24]	Y	Y	Y	PY	Y	Y	Y
Puranen et al., 1996 [42]	PY	PY	Y	PY	Y	Y	Y
Puriene et al., 2011 [69]	Y	Y	Y	Y	Y	Y	Y
Sabeena et al., 2016 [43]	Y	Y	Y	Y	Y	Y	Y
Sadaksharam et al., 2019 [44]	Y	Y	Y	Y	Y	Y	Y
Sarraj et al., 2013 [70]	Y	Y	Y	Y	Y	Y	Y
Saunders et al., 2010 [71]	Y	Y	Y	Y	Y	Y	Y
Sinclair et al., 2005 [25]	Y	PN	Y	Y	Y	Y	Y
Squires et al., 1999 [45]	Y	Y	PY	PY	Y	Y	PY
Starink et al., 1977 [72]	Y	Y	Y	Y	PN	Y	Y
Swan et al., 1981 [46]	Y	Y	PY	Y	PN	Y	Y
Tan et al., 1995 [73]	Y	Y	Y	Y	Y	Y	Y
Wadhera et al., 2012 [47]	Y	Y	Y	Y	Y	Y	Y
Wallace et al., 1976 [74]	Y	Y	Y	Y	Y	Y	Y
Yasar et al., 2009 [75]	Y	Y	Y	PY	Y	PY	PY
Yospe et al., 1995 [48]	Y	Y	Y	Y	Y	Y	Y

## Data Availability

Data are available in MEDLINE/PubMed, Scopus, and BioMed Central databases.
